# Methyl methacrylate and respiratory sensitization: A Critical review

**DOI:** 10.3109/10408444.2010.532768

**Published:** 2011-03-14

**Authors:** Jonathan Borak, Cheryl Fields, Larry S Andrews, Mark A Pemberton

**Affiliations:** 1 Departments of Epidemiology and Public Health; 2 Medicine, Yale University, New Haven, Connecticut, USA; 3 The Dow Chemical Company, Spring House, Pennsylvania, USA; 4 Lucite International UK Ltd., Billingham, United Kingdom; 5 Jonathan Borak & Company, New Haven, Connecticut, USA

**Keywords:** Acrylates, asthma, cross-reactivity, epidemiology, exposure assessment, in chemico, in silico, LLNA, mechanisms, metabolism, mixtures, occupational respiratory irritation, toxicology

## Abstract

Methyl methacrylate (MMA) is a respiratory irritant and dermal sensitizer that has been associated with occupational asthma in a small number of case reports. Those reports have raised concern that it might be a respiratory sensitizer. To better understand that possibility, we reviewed the in silico, in chemico, in vitro, and in vivo toxicology literature, and also epidemiologic and occupational medicine reports related to the respiratory effects of MMA. Numerous in silico and in chemico studies indicate that MMA is unlikely to be a respiratory sensitizer. The few in vitro studies suggest that MMA has generally weak effects. In vivo studies have documented contact skin sensitization, nonspecific cytotoxicity, and weakly positive responses on local lymph node assay; guinea pig and mouse inhalation sensitization tests have not been performed. Cohort and cross-sectional worker studies reported irritation of eyes, nose, and upper respiratory tract associated with short-term peaks exposures, but little evidence for respiratory sensitization or asthma. Nineteen case reports described asthma, laryngitis, or hypersensitivity pneumonitis in MMA-exposed workers; however, exposures were either not well described or involved mixtures containing more reactive respiratory sensitizers and irritants.The weight of evidence, both experimental and observational, argues that MMA is not a respiratory sensitizer.

## Introduction

Methyl methacrylate (MMA; C_5_H_8_O_2_; CAS No. 80-62-6) is an α,β-unsaturated ester monomer that is produced in high volumes and used widely to make polymers employed in a wide range of industrial and consumer applications, including transparent impact-resistant plastic sheets (e.g., Plexiglas®, Lucite®), dental and surgical cements, surface coatings, and injection molding and extrusion. MMA has sometimes been viewed as an “innocuous substance” (Bratt and Hathway, 1977; Elovaara et al., 1983) because of its very high reported LD_50_ value (Tansy et al., 1980) and in vitro cytotoxicity that is substantially lower than that of other acrylate and methacrylate monomers (Fujisawa et al., 2000; Geurtsen, 2000; Schweikl et al., 2001).

In contrast to its relative lack of lethality, MMA is a recognized irritant and skin sensitizer. It was reported that MMA can cause “severe” skin and respiratory irritation, and it has mild dermal sensitizing potential in animal studies (ECETOC, 1995; EU, 2002; IPCS, 1998). Both dermal sensitization and respiratory irritation have also been reported in workers and others exposed to MMA. Of greater potential concern is the possibility that MMA acts as a human respiratory sensitizer. Because of the large numbers of workers and consumers who might be exposed to MMA, the potential burden of MMA-induced asthma could be considerable. However, that possibility has proven difficult to confirm in humans.

Despite large numbers of exposed workers, only a small number of cases have been reported that seemingly link MMA with workplace asthma. Most of those cases involved mixed exposures: MMA is rarely encountered without additives (e.g., stabilizers, accelerators, and/or antibiotics) and it is often used in mixtures with other acrylate and methacrylate monomers. In addition, it can be difficult to distinguish asthma induced by respiratory sensitizers from that due to respiratory irritants (Banks and Jalloul, 2007; Kimber et al., 2007; Tarlo et al., 2008; Tarlo and Broder, 1989; Tarlo and Malo, 2009). Thus, it remains uncertain whether MMA poses significant risks of respiratory sensitization and asthma.

Recent comprehensive reviews found “no convincing evidence” that MMA was a respiratory sensitizer in humans (EU, 2002; IPCS, 1998), but the most recent (published in 2002) relied on a literature search performed in 1995. Accordingly, it seemed appropriate to update that literature search and critically review the evidence regarding MMA and respiratory sensitization versus respiratory irritation.

## Exposures and exposed populations

MMA is a widely used, high-volume synthetic chemical. Global production, estimated to be 1.4 million metric tons in 1988 (IPCS, 1998), has expanded to an estimated annual capacity greater than 2.5 million metric tons (>5.5 billion lbs) (Nexant, 2006). In 1992, US production was estimated to be nearly 1.1 billion lbs (US EPA, 1994); since then US production has grown. There are also large and growing numbers of workers potentially exposed to MMA. In the National Occupational Exposure Survey (NOES), the National Institute for Occupational Safety and Health (NIOSH) found that during 1981-1983 there were 170,000 US workers exposed to MMA, nearly twice the 90,000 workers estimated to have been exposed during 1972-1974 (NIOSH, 1990; Young, 2010).

To better understand the nature of these worker exposures, it is useful to divide industry sectors and their workers into primary versus secondary MMA users. The former includes production of MMA, MMA polymer (pMMA) and methacrylate resins, as well as extrusion and casting of methacrylate sheets (e.g., Plexiglas®, Lucite®).

These activities, performed in large industrial plants, currently employ more than 15,000 workers worldwide (Pemberton, 2010). The secondary users, who represent the great majority of exposed workers, are comprised of a much larger number of generally small, nonindustrial facilities, particularly in health care and cosmetology. MMA-containing materials are important components of orthopedic bone cement, dental composites, and cosmetic products used to sculpt and enhance fingernails.

The precise number of workers actually exposed to MMA in secondary industries is uncertain, but the number is very large. NIOSH reported that about 25,000 US health care workers were exposed to MMA in 1981-1983 (NIOSH, 1990), and that number has greatly expanded. Consider, for example, that the number of hip replacement surgeries more than doubled worldwide from the 1980s to the 1990s, and then redoubled over the next decade (Espehaug et al., 2006; Katz, 2006; Kiefer, 2007; Malchau et al., 2005). Currently more than 200,000 total hip replacements are performed annually in the USA and more than 600,000 are performed annually in Europe. Likewise, between 1979 and 2009, the number of US dentists grew from 161,000 to about 250,000, whereas the number of US dental assistants increased from 129,000 to >400,000 (ADA, 2007, 2008; BLS, 2010; Kaiser, 2010). During that time, acrylate composites were increasingly adopted in place of amalgam fillings; a 2008 European Commission report described “hundreds of millions” of dental restorations performed annually (SCENIHR, 2008). As for cosmetology, there were more than 155,000 manicurists and pedicurists in the USA in 2007 (US EPA, 2007). In 1974, US Food and Drug Administration (FDA) banned nail products containing 100% MMA monomer (US FDA, 2010), but no regulations specifically prohibit the use of MMA monomer at lower concentrations in cosmetic products and it continues to be found in nail products in the USA and many other countries (NICNAS, 2009; Work Safe Alberta, 2009).

Levels of MMA exposure differ widely across these various industries. As might be expected, the large facilities of primary MMA users have been most systematically assessed. Those workers generally had time-weighted average exposures at or below the current Occupational Safety and Health Administration (OSHA) occupational exposure limit of 100 ppm, but older reports described short-term exposures of 180 ppm or more (CEFIC, 1995; Cromer, 1976; Della Torre et al., 1982; Mizunuma et al., 1993; Monroe, 1981; Pausch et al., 1994; Pickering et al., 1993; Tomenson et al., 2000; Vos and Stephens, 2008). By contrast, much higher exposures have been reported among secondary users. For example, daily mean concentrations up to 600 ppm were reported in workers who applied MMA-containing floor coatings (CEFIC, 1995; Lindberg et al., 1991). Short-term exposures of 200 to >700ppm have been detected while bone cement was being prepared in an operating room (Darre et al., 1987; McLaughlin et al., 1979; Pickering et al., 1986). In general, however, exposures have not been systematically studied in secondary user industries.

## Respiratory sensitization versus respiratory irritation

*Respiratory sensitization* is an immunological state of the respiratory tract that results from specific adaptive immune responses to antigenic exposure, leading to heightened immunological responsiveness after subsequent exposures to the sensitizing antigen. In turn, such heightened respiratory tract responsiveness can result in allergic reactions characterized by airway obstruction, nonspecific bronchial hyperreactivity, and inflammation that may present clinically as allergic rhinitis, asthma, and extrinsic allergic alveolitis ("hypersensitivity pneu-monitis") (Boverhof et al., 2008; Isola et al., 2008; Kimber et al., 2007). Agents that provoke such immune response are referred to as *respiratory sensitizers,* i.e., “a substance that will induce a state of hypersensitivity of the airways following inhalation of the substance” (UN, 2007a).

Low-molecular-weight (LMW) respiratory sensitizers share properties with the larger class of contact sensitizers, but their specific physiological effects result from mechanistically different processes, and they are much fewer in number. Not more than about 40 LMW respiratory sensitizers have been recognized, in contrast to greater than 500 contact skin sensitizers (Vandebriel and van Loveren, 2010). The activity of both classes depends on their ability to form stable immunogenic complexes with proteins; their bioavailability, which allows them to reach epithelial tissues, engage dendritic cells, and be effectively presented to T lymphocytes; and their ability to induce production of cytokines that stimulate and differentiate immunological reactions (De Jong et al., 2009; Kimber and Dearman, 2005). In addition, both classes of sensitizers test positively on standard skin sensitization assays. For such reasons, chemicals identified as contact sensitizers are often suspected of posing a potential for respiratory sensitization. On the other hand, contact sensitization and respiratory sensitization are different hypersensitivity phenomena caused by differing immunological mechanisms (Enoch et al., 2009; Kimber and Dearman, 2005; Rodford et al., 2003). Respiratory and contact sensitizers induce different cytokine profiles, provoke responses by different T-cell populations, and most respiratory sensitizers (but not contact sensitizers) induce specific immunoglobulin E (IgE) (De Jong et al., 2009; Kimber et al., 2010; Toebak et al., 2006; Vandebriel and van Loveren, 2010). The implications of these differences and the tests and assays used to identify and characterize respiratory sensitizers are discussed below.

*Respiratory irritation,* by contrast, is a nonimmu-nological state of the respiratory tract that results from inhalation of irritant substances at doses sufficient to cause inflammation. Such irritation maybe mediated by neural reflexes (e.g., “sensory irritation") or cytotoxicity (Alarie, 1973b; Nielsen, 1991). Respiratory irritants can cause syndromes that are clinically similar to those that result from respiratory sensitization and it can be difficult clinically to determine that an individual suffers sensitizer- versus irritant-induced respiratory disease. For that reason, pulmonary physicians speak of *occupational asthma* (OA), a category that includes both sensitization and irritation (Bernstein et al., 2006b; Francis et al., 2007; Malo and Newman-Taylor, 2007; Tarlo et al., 2008); studies of OA do not often distinguish between hypersensitivity and irritant causes (Nicholson et al., 2005). From a clinical perspective, such an approach is reasonable because treatments for both are similar and because sensitizers cause the great majority of OA, especially in well-controlled work sites where high-level irritant exposures are rare (Gautrin et al., 2006; McFadden and Gilbert, 1992; Nicholson et al., 2005).

However, that approach provides little information about the etiology and mechanisms of disease. Such information is important for selecting appropriate prevention and control practices, which differ for sensitizers and irritants. In most cases, irritant effects can be avoided using industrial engineering and hygiene controls that reduce exposures to “safe” levels. By contrast, there are generally no “safe” exposure levels for agents to which workers and others have been sensitized; avoidance of further exposures often requires a change in job function or occupation. Moreover, respiratory sensitizers are regulated to a higher standard of safety and control under REACH (Registration, Evaluation, Authorisation and Restriction of Chemicals) and other regulatory schemes than respiratory irritants.

Because respiratory sensitization is widely regarded as an adverse effect of much concern and substantial morbidity, and in order to implement necessary prevention and control procedures, considerable efforts have been made to develop methods to identify and characterize substances that act as respiratory sensitizers, with particular concerns for workplace chemicals. It is estimated that about 15% of adult asthma is attributable to occupational factors (Balmes et al., 2003; Beach et al., 2005; Tarlo et al., 2008) and up to 90% of those cases result from respiratory sensitization (McFadden and Gilbert, 1992; Nicholson et al., 2005). However, there has been particular difficulty identifying and characterizing the respiratory sensitization capacity of low-molecular-weight (LMW; <1kDa) compounds, in part due to persistent uncertainty about the immunological mechanisms by which these agents induce respiratory hypersensitivity (Boverhof et al., 2008; Isola et al., 2008; Kimber et al., 2007). Identification of respiratory sensitizers is also challenging because, as noted above, the syndromes caused by sensitizers and irritants can be very similar.

Evaluations of the respiratory toxicity of MMA have faced the same challenges. Numerous studies have documented its capacity to cause contact skin sensitization, whereas others have documented MMA-induced irritation of the skin and respiratory tract, but only occasional reports suggest that MMA might also be a respiratory sensitizer. The extensive toxicology literature on MMA includes in silico studies, in chemico studies, in vitro studies, in vivo animal studies, and a variety of human observational studies that range from cohort studies to isolated case reports. The following sections of this report first describe the metabolism and mechanisms of action of MMA and then review the accumulated toxicology literature grouped according to study types.

## Metabolism and mechanisms of action

**Figure 1:**
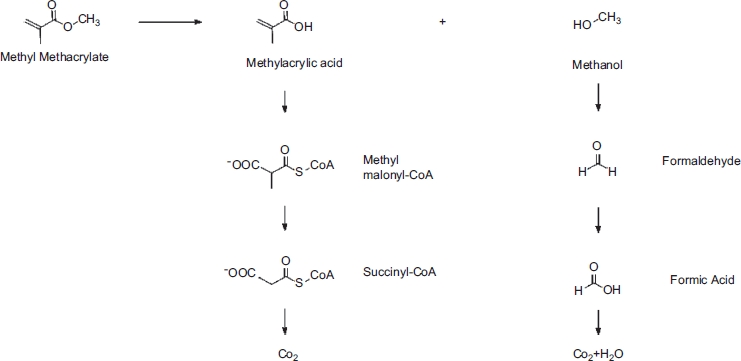
Metabolism of methyl methacrylate (courtesy of Peter Blomgren).

The toxicity of MMA, particularly its capacity to cause irritation and to induce sensitization, is directly related to its metabolism and reactive chemistry. The principal metabolic pathway for MMA entails ester hydrolysis to methacrylic acid and methanol (Figure 1), a process catalyzed by carboxylesterase (Greim et al., 1995). In turn, both intermediary metabolites are further metabolized to carbon dioxide and water. Methacrylic acid is sequentially transformed to methyl malonyl-coenzyme A (CoA) and succinyl-CoA, which enters the citric acid cycle and is thereby oxidized (Bratt and Hathway, 1977; Crout et al., 1982). Methanol is transformed via formic acid to carbon dioxide.

Hydrolysis and further metabolism of MMA occur rapidly following exposure. In rats administered C^14^-labeled MMA orally (5.7 or 120mg/kg) or intravenously (5.7 or 6.8mg/kg), about 65% of the radioactivity was expired within 2 hours and 84-88% within 10 days; pulmonary excretion of unchanged MMA accounted for less than 1.4% (Bratt and Hathway, 1977). Following intraperito-neal injection of C^14^-labeled MMA (35 or 45mg/kg) in rats, 72% of the radioactivity was expired within 4 hours (Crout et al., 1982).

Such in vivo metabolic studies have notbeen described in humans, but in vitro studies of liver cells and olfactory and respiratory cells obtained from the nasal tracts of humans, rats, and hamsters have demonstrated qualitatively similar, but quantitatively different, across-species carboxylesterase activity for MMA (Mainwaring et al., 2001). In those studies, human tissue was insufficient for determination of individual metabolic rate constants, but comparisons of pooled microsomal fractions indicated that human liver had a greater *V*_max_ than rat or hamster livers (*V*_max_: 494 versus 46.5 versus 137 nmol/min/mg protein), whereas the corresponding activity of human respiratory cell microsomes was lower (V_max_: 2.7 versus 14.3 versus 3.6 nmol/min/mg protein). Comparisons between human and rat olfactory cell S9 fractions revealed still greater interspecies differences (V_max_: 0.48 versus 12 nmol/min/mg protein).

The relevance of olfactory and respiratory carboxylesterase activity to MMA-induced respiratory irritation and cytotoxicity has also been demonstrated. In animal studies, high level MMA inhalation exposure was cytotoxic to nasal epithelium, leading to inflammatory cell infiltration, degeneration, atrophy, and metaplasia (Chan et al., 1988; Hext et al., 2001; Mainwaring et al., 2001), effects caused by methacrylic acid-induced irritation. Inhibition of enzymic activity by bis(4-nitrophenyl) phosphate (BNPP), a carboxylesterase inhibitor, significantly reduced the extent and severity of epithelial injury (Mainwaring et al., 2001). BNPP-induced carboxylesterase inhibition also significantly reduced deposition of inhaled MMA in the upper respiratory tract of anesthetized rats (Morris, 1992; Morris and Frederick, 1995). Thus, both respiratory tract dose and cytotoxicity following inhalation exposure are determined by carboxylesterase activity. In a similar manner, the importance of carboxylesterase-mediated hydrolysis for the metabolism of related acrylate and methacrylate esters has also been demonstrated (McCarthy and Witz, 1997).

A second important metabolic pathway for MMA involves reaction with tissue nucleophiles via Michael addition on the electrophilic C of the a,β-unsaturated carboxyl group (Freidig et al., 1999; Greim et al., 1995; McCarthy et al., 1994). The prototype for such reactions is conjugation with glutathione (GSH), which occurs spontaneously and enzymatically, leading to formation of thioethers and mercapturic acids. Increased urinary excretion of thioethers and depletion of hepatocyte GSH have been documented following in vivo and in vitro exposures to MMA and other acrylate and methacrylate esters (Delbressine et al., 1981; Elovaara et al., 1983).

The electrophilic reactivity of low-molecular-weight molecules, as reflected by their interactions with glutathione and other nucleophiles, is an important aspect of their ability to act as sensitizers (Enoch et al., 2008, 2009, 2010; Roberts et al., 2007, 2008; Smith and Hotchkiss, 2001). In skin sensitization studies, a key early step in the process leading to sensitization is the formation of covalent adducts with a carrier protein, thereby forming an antigenic hapten-protein complex (Natsch and Emter, 2008; Roberts et al., 2008; Roberts and Aptula, 2008; Smith and Hotchkiss, 2001). Such covalent protein binding has also been described as an “essential step” required for respiratory sensitization (Enoch et al., 2009, 2010) and electrophilic reactivity is said to predict sensitization potential, although it is not the only important determinant. Within mechanistic domains and sub categories of electrophiles, the ability of compounds to cause respiratory sensitization has been related to their relative elec-trophilicity. Michael acceptor electrophiles, such as MMA and related esters, are predicted to be strong sensitizers (Natsch and Emter, 2008; Roberts et al., 2007a), but there is a broad spectrum of electrophilic reactivity across those esters (see Table 1 and discussion below). However, electrophilicity alone cannot be used to directly compare sensitization potential across mechanistic categories and subdomains (e.g., Michael acceptors versus acylators versus S_n_Ar) (Enoch et al., 2009, 2010).

**Table 1:** Reactivity of MMA and other representative (meth)acrylates, respiratory sensitizers and irritants, dermal sensitizers, and nonsensitizers.

			Measures of electrophilicity			
Chemicals	Mechanistic category	LLNA EC3 (↑value→↓ reactivity)	Electrophilic Index, ω (↑value→↑ reactivity)	GSH depletion, *K*_GSH_ (L mol^-1^ min^-1^) (↑value→↑ reactivity)	GSH depletion, RC_50_ (mM) (↑value→↓ reactivity)	Log *P*
**Methyl methacrylate and other (meth) acrylates**						
Methyl methacrylate	Michael addition	60–90	0.85(9)	0.20(12)	70(7)	1.38
		“Weak”(1)		0.33(13)	74.1 (15)	
				6(14)	76(17)	
Methyl acrylate	Michael addition	19.6	1.02(9)	11.4(15)	0.42(15)	0.8
		“Weak”(2)	52.0(13)			
				61(14)		
Ethyl methacrylate	Michael addition	No data	0.83(9)	0.058(15)	NRAS(7, 17)	1.94
				0.139(13)		
				4(14)		
Ethyl acrylate	Michael addition	28	0.99(9)	10.6(15)	0.52(7, 17)	1.32
		“Weak”(3)		11.9(16)		
				26.6(13)		
				39.7(12)		
				57(14)		
Allyl methacrylate	Michael addition	No data	No data	0.51(12)	No data	2.12
				3(14)		
Allyl acrylate	Michael addition	No data	0.94(9)	19.5(9)	3.5(17)	1.4
2-Hydroxypropyl methacrylate	Michael addition	“Non-sensitizer” (3)	No data	4(14)	28(7)	0.97
2-Hydroxyethyl methacrylate	Michael addition	“Non-sensitizer” (4)	No data	33(8)	No data	0.47
2-Hydroxyethyl acrylate	Michael addition	1.4	No data	22.2(16)	0.27(7)	−0.21
		“Moderate” (3)		50.9(12)		
				102(14)		
**Representative respiratory sensitizers**						
Trimellitic anhydride	Acylation	0.22	2.35(10)	No data	No data	1.95
		“Strong” (5)				
2,4-TDI	Acylation	0.11	1.37(10)	No data	No data	3.74
(toluene diisocyanate)		“Strong” (5)	1.23(11)			
Phthalic anhydride	Acylation	0.36	2.61(11)	No data	No data	1.6
		“Strong” (5)				
**Known or suspected respiratory sensitizers**						
Formaldehyde	Acylation	0.4	1.46(11)	No data	No data	0.35
		“Strong” (5)				
Hydroquinone	Michael Addition	0.11	No data	No data	4.4(7)	0.59
		“Strong” (3)				
**Representative respiratory irritant**						
Acrolein	Michael addition	0.86	No data	No data	0.086(17)	−0.01
		“Strong” (8)				
**Representative contact sensitizer**						
DNCB	S_n_Ar	0.04	3.32(11)	No data	No data	2.17
(dinitrochlorobenzene)		“Extreme” (1)				
**Representative non-irritant, non-sensitizer**						
Methyl tiglate	Michael addition	No data	1.24(10)	0.007(15)	NRAS(15, 17)	1.69
			0.694 (9)			
		(1) Betts 2006	(9) Wondrousch 2010	(9) Wondrousch 2010	(7) Schultz 2009	(18) NLM 2010
		(2) Dearman 2007	(10) Enoch 2010	(12) Freidig 1999	(15) Bohme 2009	
		(3) Gerberick 2005	(11) Enoch 2009	(13) McCarthy 1994	(17) Yarbrough 2007	
		(4) Roberts 2007a		(14) Chan 2008		
		(6) Gerberick 2007b		(15) Bohme 2009		
		(7) Schultz 2009		(16) Roberts 2009		
		(8) Schultz 2007				

Key: NRAS: = not reactive at saturation.

Hydrolysis of MMA reduces its sensitization potential because, under physiological conditions, methacrylic and acrylic acids are not electrophilic or protein reactive (Frederick and Reynolds, 1989; Smith and Hotchkiss, 2001). Inratstudies, intraperitoneal(IP) administration of 0.14 mM/kg (∼ 14 mg/kg) MMA did not result in increased urinary excretion of thioethers, whereas thioether excretion was significantly increased in animals pretreated with a carboxylesterase inhibitor (tri-o-tolyl phosphate) (Delbressine et al., 1981). In contrast to MMA, administration of comparable doses of methyl acrylate resulted in demonstrable thioether excretion both with and without carboxylesterase inhibition. These findings suggest that hydrolysis is the principal pathway of MMA metabolism, that electrophilic reactions via Michael addition play only a minor role, and that such reactions occur only at high tissue concentrations (Greim et al., 1995).

## In silico analyses

### Background

Considerable effort has been made to identify LMW sen-sitizers and characterize their sensitization potential by means of qualitative and quantitative structure-activity relationships (SARs) and SAR analyses. SAR analyses use computational methods to identify submolecular structural features (i.e., molecular fragments, functional groups) that have been associated with specific biological effects, either subcellular effects, such as protein reactivity and up-regulation of specific cytokines, or clinical effects such as provocation of asthma (Gerberick et al., 2008; Graham et al., 1997; Patlewicz et al., 2007). Submolecular features identified in this way are referred to as “structural alerts.” The presence and pattern of particular structural alerts provide a basis for predictions that specific molecules cause particular biological effects. Such analyses are computer intensive, utilizing artificial intelligence software (i.e., “expert systems") to evaluate large data sets containing the physical properties, chemical properties, and biological activities of the chemicals under analysis.

A number of SAR expert systems have been developed and used to compare the molecular structures of LMW chemicals with known sensitizing or irritancy activity. An example is the relative alkylation index (RAI), which estimates a compound's relative degree of covalent binding with host carrier proteins (Roberts et al., 2007a, 2009). RAI is based on the understanding that sensitization depends quantitatively on the degree of such binding, which can be estimated by the rate constant for the reaction of the compound with specific nucleophiles. As described in following sections, various in silico, in chemico, and in vitro assays have been used to determine the rate constants for these analytical models. By comparing RAI values of 106 LMW compounds with their corresponding in vivo local lymph node assay (LLNA) results, six “major reaction mechanistic domains” were identified that served to predict the relative sensitization potency for 87 of the compounds (Roberts et al., 2007a). A related approach employed the electrophilic index (co) to estimate the sensitization potential of 19 LMW chemicals characterized as Michael acceptors (Enoch et al., 2008). Results showed “good agreement” with those of LLNA, but the sensitivity, specificity, and predictive values of the methods were not described.

A more clinical example of the uses of SAR was provided by five analyses that used different software to analyze different databases to compare the molecular structures of LMW chemicals known to cause asthma and other compounds known to not cause asthma. Although these five analyses employed different statistical protocols, analyzed different chemical databases, and identified differing numbers and types of structural alerts, their results were surprisingly similar (Kimber et al., 2007; Seed et al., 2008). The sensitivity of the expert systems for identifying the respiratory sensitizers ranged from 85% to 96%, whereas their specificity ranged from 74% to 99% (Cunningham et al., 2005; Graham et al., 1997; Jarvis et al., 2005; Karol et al., 2001). In almost every case, the expert systems correctly identified compounds that were not respiratory sensitizers, yielding an apparent negative predictive value (NPV) >95%. On the other hand, each system failed to identify at least some of the respiratory sensitizers. To calculate the positive predictive value (PPV) of these methods, it is necessary to know not only their sensitivity and specificity, but also the proportion of all chemicals that are actually respiratory sensitizers, i.e., the prior probability. If the proportion of respiratory sensitizers was 1:30, then the PPV of those five systems would have ranged from 0.10 to 0.75, but if only 1 in 300 chemicals was a respiratory sensitizer, the PPV would been only 0.01 to 0.22 (Seed et al., 2008). Because only a limited number of LMW respiratory sensitizers have been identified to date, it seems likely that the true prior probability will prove relatively small, thus implying a small PPV for the method.

SAR analysis has been proposed for initial screening of potential sensitizers. An extensive literature documents its utility for contact sensitizers, but there is only limited experience for respiratory sensitizers and irritants (Gerberick et al., 2008; Patlewicz et al., 2007; Rodford et al., 2003). Because of their high NPV, SAR analyses seem able to effectively exclude from further testing LMW chemicals that are not respiratory sensitizers. But because of their relatively low PPV, SAR analyses alone are probably not sufficient to conclude that a specific agent is a respiratory sensitizer.

### In silico analyses of MMA

#### Electrophilic reactivity

The electrophilicity of MMA has been evaluated using two different in silico analytical systems. One approach predicted the electrophilic index (ω) by modeling the rate constants of specific electrophile-nucleophile reactions based on their ionization potential and electron affinity, which are quantified in terms of their highest occupied molecular orbital (HOMO) and lowest unoccupied molecular orbital (LUMO) energies (Enoch et al., 2009, 2010; Wondrousch et al., 2010). Increasing values of ω indicate increasing reactivity with nucleophiles. The second approach employed nuclearmagnetic resonance (NMR) spectroscopy to characterize the electron density of the β-carbon of the (meth)acrylate α-β-double bond (Fujisawa and Kadoma, 2009; Ishihara and Fujisawa, 2009). The magnitude of NMR shift correlates with electrophilic reactivity. For example, high correlation (r^2^ = .998) was found between NMR results of 20 acrylates and methacrylates and their corresponding glutathione reactivity (Fujisawa and Kadoma, 2009).

Table 1 presents the electrophilic index (ω) of MMA and selected acrylates, methacrylates, known respiratory and contact sensitizers, a representative respiratory irritant, and a representative nonsensitizer, nonirritant. It can be seen that the electrophilic index (ω) values of MMA and other methacrylates were lower than corresponding values of acrylates and much lower than those of known respiratory sensitizers.

#### Structure-activity relationships

Schultz and colleagues (Schultz et al., 2007, 2009; Yarbrough and Schultz, 2007) used a combination of in chemico glutathione reactivity measures and in vivo LLNA data to identify reactive structural fragments within the subcategories of Michaels acceptors. They characterized 10 subcategories; MMA and other methacrylates were grouped into the subcategory associated with slow rates of reaction and weak sensitizing potential. They attributed the lack of reactivity and sensitizing potency to the α-methyl substitution of the α-β-double bond.

Patlewicz et al. (Patlewicz et al., 2007) evaluated the performance of three expert SAR models developed to identify contact skin sensitizers, not respiratory sensitizers, using a database of 211 LMW chemicals previously tested by LLNA (Gerberick et al., 2005). MMA was not included in the database, but there were other acrylate and methacrylate esters. The authors concluded that although the α-β-double bond fragment of the methacrylate structure is electrophilic and can act as a Michael acceptor, the presence of α-methyl substitution “destabilizes the Michael transition state” and significantly reduces its capacity to sensitize. This finding supports the conclusion by McCarthy et al. (McCarthy et al., 1994), who evaluated a different set of LMW chemicals that did include MMA.

Several other studies performed qualitative SAR analyses to determine the types and numbers of functional groups found on the molecules of LMW industrial chemicals that had been documented to cause respiratory sensitization and/or human asthma. Although not all of the following analyses specifically consider MMA, their findings are relevant. Agius and colleagues (Agius et al., 1991, 1994; Agius, 2000; Seed and Agius, 2010) reviewed the literature on occupational asthma to identify LMW industrial chemicals with sufficient clinical or epidemiological evidence indicating that the chemical caused asthma. For those chemicals, the types and frequency of functional molecular subgroups were determined and compared to the frequency of such groups for chemicals believed to not cause asthma. A major finding was that the likelihood of a LMW chemical causing respiratory sensitization and asthma increased significantly if it had at least two reactive groups that could lead to protein cross-linking (Seed et al., 2008), whereas monofunctional molecules (e.g., MMA) posed smaller risks.

Graham et al. (Graham et al., 1997) identified 40 respiratory sensitizers, i.e., LMW chemicals documented to elicit a decrease in forced expiratory volume in one second (FEV1) >20% within 24 hours of provocation challenge, which were nonmetallic and contained at least two contiguous nonhydrogen atoms. Two software systems were used to compare the molecular structures of those chemicals with the structures of 120 LMW chemicals not known to be sensitizers. The analyses and resulting SAR model were validated by means of a reiterative data withholding exercise. There were 16 molecular fragments ("biophores") associated with the active chemicals (12 with *p* <.O5) and four fragments ("biophobes") associated with the inactive chemicals (three with *p* <.O5). No fragment of MMA was on the list of biophores, but esters (CO-O-CH2-) were on the list of biophobes (p <.001). Model validation indicated that sensitivity and specificity were both >95%.

Jarvis et al. (Jarvis et al., 2005) performed SAR analysis on 78 LMW chemicals reported to cause asthma in humans and 301 LMW industrial chemicals not known to be respiratory sensitizers. Logistic regression was used to determine the statistical associations between the asthmagens and 25 categories of molecular fragments. In light of those statistical associations, a “hazard index” was calculated that predicted whether each chemical was an asthma hazard. The SAR model was then validated on a second set of LMW chemicals that included 21 known asthmagens, 16 suspected asthmagens, and 77 nonsen-sitizers. For the validation set, the model had a sensitivity of 86% and a specificity of 99%. MMA was one of the chemicals included in the analysis; it was classified as “inactive,” i.e., it was predicted to not be an occupational asthma hazard.

### Section summary

A limited number of SAR studies have considered the contact and respiratory sensitization potential of MMA. Two groups of studies found that a methyl group on the α-carbon of the acrylate double bond substantially reduced sensitizing capacity and that the electrophilic reactivity of MMA was significantly less than corresponding acrylate esters. Three studies reported that the respiratory sensitization hazard of LMW asthmagens was substantially increased if they had at least two reactive functional groups; MMA has only one, the α-methyl substituted double bond. Another study of chemicals associated with occupational asthma characterized esters as nonsensitizers. Finally, one SAR analysis with a model specificity of 99% concluded that MMA was “inactive” and not a respiratory sensitizer.

Although the number of SAR studies is limited, they provide a weight of evidence that MMA does not cause respiratory sensitization. However, the role of SAR analyses for the identification of sensitizers remains limited; it is currently not sufficient to be regarded as “a standalone tool for hazard identification” (Patlewicz et al., 2007).

## In chemico analyses

### Background

In chemico analyses can be used to characterize the properties of LMW chemicals that impact their ability to react with proteins and other nucleophiles. One commonly used assay type measures the disappearance of a nucleophile mixed with the LMW chemical and/or the formation of adducts between that LMW chemical and the nucleophile (Vandebriel and van Loveren, 2010). Such assays yield quantitative data that are often reported as either of two related terms, RC_50_ or *K*_GSH_ (Roberts et al., 2008; Vandebriel and van Loveren, 2010). The RC_50_ is the concentration of electrophile required to deplete 50% of a fixed quantity of a sulfhydryl-containing compound (e.g., GSH) in a fixed time period (Chipinda et al., 2010). The *K*_GSH_ is the apparent rate constant for such a reaction with glutathione (Bohme et al., 2009). More reactive chemicals have smaller RC_50_ and larger K_GSH_ values than less reactive chemicals.

A related in chemico approach to characterize the reactivity of LMW compounds is the peptide reactivity assay (PRA), which measures the depletion of GSH or other synthetic mononucleophilic peptides after treatment with an excess of electrophile (Aleksic et al., 2009; Gerberick et al., 2004, 2007b, 2008; Roggen et al., 2008). Results of PRA can be compared to estimates of sensitization potency derived from other tests, such as in vivo LLNA (Aleksic et al., 2009; Gerberick et al., 2004; Mutschler et al., 2009). Predictive accuracy for contact sensitizers up to 90% has been reported for PRA, but its predictive accuracy for respiratory sensitizers has not been determined. Published reports have not described use of PRA to differentiate respiratory sensitizers, contact sensitizers, and respiratory irritants (Boverhof et al., 2008; Gerberick et al., 2008; Kimber et al., 2007; Roggen et al., 2008).

Hydrophobicity (i.e., logP), another property of LMW chemicals that impacts their ability to covalently bond with the nucleophile groups of carrier proteins, has also been evaluated using in chemico analyses (Roberts et al., 2007; Vandebriel and van Loveren, 2010). It has been estimated that sensitization potential is about twice as dependent on electrophilic reactivity as hydrophobicity (Roberts et al., 2008). It has also been shown that the cytotoxicity of acrylate and methacrylate esters was directly related to their lipid solubility (r^2^ of .718 and .950, respectively) (Yoshii, 1997).

### In chemico analyses of MMA

#### RC_50_ and K_GSH_ assays

The RC_50_ values for MMA and a variety of other LMW chemicals were determined by Schultz and colleagues (Schultz et al., 2009; Yarbrough and Schultz, 2007) using a spectrophotometric assay to measure sulfhydryl group depletion following 2-hour reactions with GSH. They evaluated 83 Michael acceptor chemicals, including 10 acrylates, six methacrylates, and one dimethacrylate. Bohme et al. (Bohme et al., 2009) used a modification of that method to determine RC_50_ values for 26 Michael acceptors, including MMA and five other (meth) acrylates.

The *K*_GSH_ for various electrophilic Michael acceptors have been calculated by measuring the rates of GSH depletion under controlled conditions. Freidig et al. (Freidig et al., 1999) evaluated six acrylate and seven methacrylate esters including MMA. Esters were incubated with GSH at 20°C and pH 8.8 for 1 hour (acrylate esters) or 24 hours (methacrylate esters). GSH depletion was measured by high-performance liquid chromatog-raphy (HPLC). Chan and O'Brien (2008) incubated five acrylates and five methacrylates, including MMA, with GSH at 25°C and pH 8.0 and measured the remaining GSH by spectrophotometry at various times from 10 to 180 minutes. Bohme et al. (2009) and Wondrousch et al. (2010) incubated 26 Michael acceptors, including MMA and five other (meth)acrylates, with GSH at 25°C and pH 7.4 and measured GSH depletion and oxidation using Ultraviolet-visible spectroscopy (UV-VIS) at up to eight time points per reaction. Roberts and Nash (Roberts and Natsch, 2009) incubated 26 LMW chemicals, including acrylates but not MMA, with a SH-based pep tide (Cor1C-420) at 25°C and pH 7.5. They used liquid chromatogra-phy-mass spectrometry (LC-MS) at 30 to 1440 minutes to measure peptide depletion and oxidation, and to characterize adduct formation.

Table 1 shows the RC_50_ and *K*_GSH_ values of MMA, other representative acrylates and methacrylates, a respiratory irritant (acrolein), and a nonirritant/nonsensitizer (methyl tiglate). It can be seen that methacrylates are less reactive than acrylates: the RC_50_ values of the methacrylates are more than 100-fold greater and the *K*_GSH_ values are 10- to 190-fold smaller than the values for the corresponding acrylates. MMA is at the low end of the reactivity range. Based solely on GSH reactivity, one would predict that methacrylate esters would be substantially less potent sensitizers than corresponding acrylates and that MMA would be among the least potent.

#### Peptide reactivity assay

In chemico PRA analyses have not been used to characterize the reactivity of MMA, although other (meth)acry-lates have been evaluated (Aleksic et al., 2009; Gerberick et al., 2007b).

#### Hydrophobicity (log P)

As seen in Table 1, the log *P* of methacrylate and acrylate esters increases as the length of their alkyl side chains increase. In addition, α-methyl substitution of the α-β-double bond increases the hydrophobicity of the ester molecules; thus the log *P* of methacrylates is about 50% greater than corresponding acrylates. MMA is among the least hydrophobic of the methacrylates. However, the increased log *P* of the methacrylates compared to acrylates is relatively small, compared to the substantially greater increased GSH reactivity of the acrylates.

### Section summary

A limited number of in chemico analyses have determined the thiol reactivity and hydrophobicity of MMA and other acrylates and methacrylate esters. Because of its relatively low electrophilic reactivity as contrasted with those other esters and relatively low log *P*, one would expect that MMA has comparatively low sensitizing potential.

## In vitro testing

### Background

Several in vitro approaches have been used to characterize the contact sensitization potential of LMW compounds. One approach, *cellular response assays,* determines the response of cultured cells incubated with specific test chemicals. Such in vitro studies can determine secreted or intracellular cytokine profiles in kera-tinocytes, dendritic cells (DCs), peripheral white blood cells, T cells, respiratory epithelial cells, and alveolar macrophages (Cameron et al., 1999; Corsini et al., 2009; Gosepath et al., 2007; Liu et al., 1999; Muller et al., 1996; Toebak et al., 2006; Vandebriel et al., 2005). Cytokines are immune system signaling proteins that are central to the stimulation and differentiation of immunological reactions. A common classification distinguishes proin-flammatory Thl-type cytokines (e.g., interleukin-2 [IL-2], interferon γ (IFN-γ), tumor necrosis factor (3) from anti-inflammatory Th2-type cytokines (e.g., IL-4, IL-5, IL-10). Thl cytokines are closely associated with enhanced cellular immune responses and contact skin sensitization, whereas Th2 cytokines favor antibody responses (Berger, 2000; Desai and Brightling, 2009; Kimber and Dearman, 2005). Respiratory sensitization is “a classical example” of Th2-mediated disease (Toebak et al., 2006).

However, such studies have been mainly used to evaluate contact sensitizers, not respiratory sensitizers. Moreover, some researchers have noted that dendritic cell responses were relatively “modest,” were “not sufficiently resilient to identify allergens,” and demonstrated considerable “donor-to-donor response variations” even when testing potent contact sensitizers (Ryan et al., 2009; Toebak et al., 2009). Accordingly, an in vitro model using such cell lines for identifying respiratory sensitizers “is currently lacking” (Vandebriel and van Loveren, 2010). By contrast, evidence suggests that IL-18, a cytokine produced in dermal (Stoll et al., 1997) and respiratory epithelium (Cameron et al., 1999), may play a useful role for identification and discrimination of contact sensitizers, respiratory sensitizers, and irritants. For example, Corsini et al. (Corsini et al., 2009) incubated human keratinocytes with one of three respiratory sensitizers, nine contact sensitizers, or four potent irritants. At noncytotoxic levels, IL-18 was induced by the contact sensitizers, but not by the respiratory sensitizers or irritants.

To date, cellular response assays have been employed mainly in experimental settings to characterize responses to a limited number of known, potent contact sensitizers, but not respiratory sensitizers. There are few published data indicating their ability to discriminate between weak sensitizers and irritants and they have apparently not been used to characterize the respiratory sensitization potential of unknown compounds.

A second approach used by some researchers is an in vitro version of the peptide reactivity assay (PRA) described above, which has more commonly been performed as an in chemico analysis.

### In vitro testing of MMA

#### Cellular response assays

Gosepath et al. (Gosepath et al., 2007) measured cytokine release and expression of corresponding mRNAs in 22 primary cell cultures containing epithelial and fibroblast cells from the inferior nasal turbinate tissue of healthy individuals. Cultures were exposed to 50 or 200 ppm MMA for 4 or 24 hours, after which cytokine and mRNA levels were determined. Controls were exposed to “synthetic air.” Levels of interleukin-1β (IL-1β), IL-6, IL-8, tumor necrosis factor a (TNFα), granulocyte-macrophage colony-stimulating factor (GM-CSF)andmonocytechemotacticprotein1(MCP-1) were measured in culture supernatant by enzyme-linked immunosorbent assay (ELISA). Quantitative polymerase chain reaction (Q-PCR) was used to measure intracellular levels of the corresponding mRNAs. Levels of mRNAs for IL-1β, IL-6, IL-8, TNFα, andMCP-1 were increased after 4-hour exposure to 50 ppm, but not 200 ppm; by 24 hours the elevated mRNA levels had regulated back to control levels. Cytokine protein levels were not increased after 4- or 24-hour exposures at either exposure level. Protein levels of the proinflam-matory cytokine IL-1β were consistently low, whereas levels of TNFα and GM-CSF actually declined during exposure. The authors concluded that MMA exposure to 50 or 200 ppm did not induce lasting up-regulation of inflammatory mediators.

Liu et al. (Liu et al., 1999) measured lymphocyte transformation and release of three cytokines, IL-6, TNFα, and IFN-y, in human whole-blood cultures from 10 healthy individuals. Cells were incubated with five MMA concentrations from 0.1 to 100 mmol/L (-10 to 10,000mg/L); cytotoxicitywas seen at levels >30 mmol/L. The cells were tested with or without stimulation by phytohemaggluti-nin (PHA) or *Staphylococcus aureus* protein A (SAP) and cytokines were measured daily for 6 days using ELISA. Compared to controls, none of the MMA exposures induced lymphocyte transformation as determined by incorporation of labeled thymidine. Release of cytokines was inconsistent. Levels of IFN-y decreased under all exposure conditions and times. Levels of IL-6 showed a nonsignificant increase in unstimulated cells, a significant increase occurred with PHA stimulation, but apparent inhibition was noted in SAP-stimulated cells. TNFα levels were increased only on day 1 in unstimulated cells, but no increase was noted in cells stimulated with PHA or SAP.

#### Peptide reactivity assay

McCarthy et al. (McCarthy et al., 1994) determined the electrophilic reactivity of MMA with GSH and compared it to the reactivity of three acrylate and three methacrylate esters (Table 1). Esters were incubated with GSH and red blood cells for 1 hour at 37°C and pH 7.4. Declines in free-sulfhydryl levels were measured as changes in optical density, and apparent rate constants were determined for the reactions of each ester with GSH. Comparisons of corresponding pairs of acrylate and methacrylate esters indicated that α-methyl substitution decreased ester reactivity toward nucleophiles. In particular, the apparent rate constant for the reaction of MMA with GSH was only 0.625% that for the reaction of methyl acrylate with GSH, indicating levels of reactivity that were substantially smaller than those of similar acrylate esters. The apparent rate constant for MMA determined by McCarthy et al. was similar to the values determined in the in chemico studies discussed above (Table 1).

### Section summary

There has been only very limited in vitro testing of MMA for the purpose of characterizing its sensitization potential. The limited published data indicate that its effects are generally weak and they provide no evidence that MMA is a respiratory sensitizer.

## In vivo animal studies

### Background

There are currently no recognized and validated in vivo test methods to identify LMW respiratory sensitizers (Basketter et al., 2009b; UN, 2007a; Vandebriel and van Loveren, 2010). The animal species most often used for respiratory sensitization testing are the mouse and guinea pig (Briatico-Vangosa et al., 1994; Karol, 1994; Pauluhn and Mohr, 2005). Both species are listed by the Globally Harmonized System of Classification and Labeling of Chemicals (GHS) as appropriate “under certain circumstances” for evaluating the relative allergenicity of high-molecular-weight (HMW) proteins (UN, 2007b), but there has been only limited experience in their use for testing LMW sensitizers. By contrast, a variety of animal models have been used to study the adverse respiratory effects of MMA exposures, but most did not specifically distinguish between respiratory irritation and respiratory sensitization. In addition, numerous studies have evaluated the capacity of MMA to induce contact skin sensitization. Summarized below are the animal studies that evaluated MMA-induced *nonspecific respiratory effects, sensory irritation, respiratory sensitization,* and *contact skin sensitization.*

### Nonspecific respiratory effects

Numerous experimental studies have evaluated the respiratory effects of acute, subchronic and chronic MMA inhalation in mice, rats, dogs, and hamsters. However, those studies did not distinguish sensitization from irritation. Most found evidence that MMA caused dose-related cytotoxicity to respiratory epithelium in the nose, trachea, and/or lung parenchyma. Observed effects, which were not species specific, included edema, inflammatory cell infiltration, degeneration, and atrophy. These studies will not be further detailed because they did not address issues of respiratory sensitization and because they have been well reviewed elsewhere (US EPA, 1998; EU, 2002; IPCS, 1998).

### Sensory irritation

"Sensory irritation” refers to a family of reflex-mediated physiological responses resulting from stimulation of trigeminal nerve endings (Alarie, 1973a; ASTM, 2004; Nielsen, 1991). The basis of sensory irritation testing ("Alarie test") is reduction of respiratory frequency caused by inhalation exposure. Most often performed in Swiss Webster mice exposed nose-only for 10 to 30 minutes, the test is “positive” if respiration frequency is reduced by 50%. The potency of a sensory irritant is typically expressed as the concentration necessary to achieve a 50% reduction in respiratory frequency (RD_50_) (Alarie etal., 1995). If increasing exposure does not result in such a 50% reduction, the agent is regarded as “not a sensory irritant,” although some toxicologists refer to slight or transient decreases in respiratory frequency as “mild sensory irritation” (Stadler, 1993). Notably, this is not a test of cytotoxicity or corrosivity (Kuwabara et al., 2007) and concerns have been raised that cytotoxic effects can occur at exposure levels that do not cause “sensory irritation” (Bos et al., 2002; Zissu, 1995).

Sensory irritation testing has been performed for MMA, methacrylic acid, and several other acrylate and methacrylate compounds. MMA and ethyl methacrylate were judged to not be sensory irritants; neither caused a 50% reduction of respiratory frequency, although each demonstrated transient “mild” effects (Stadler, 1993), and methacrylic acid was positive for sensory irritation, but demonstrated only weak potency (RD_50_: 22,000 ppm) (Stadler, 1993). By contrast, ethyl acrylate and acrylic acid were substantially more potent with RD_50_ values of 315ppm (DeCeaurriz et al., 1981) and 685ppm (Buckley et al., 1984), respectively.

These results are consistent with the physicochemical properties of methacrylates. The sensory irritant potency of individual chemicals is related to their reactivity with protein sulfhydryl groups (Alarie, 1973a, 1973b; Nielsen, 1991). As discussed above, α-methyl substitution of the unsaturated carboxyl group of methacrylates and methacrylic acid reduces their thiol reactivity as compared to the activities of corresponding unsubstituted acrylates and acrylic acid. In addition, it is likely that MMA-induced sensory irritation would depend on its hydrolysis to methacrylic acid (Nielsen, 1991), a corrosive but weak sensory irritant. Thus it is not surprising that MMA causes dose-related cytotoxicity to respiratory epithelium, but little or no sensory irritation.

### Respiratory sensitization

A number of in vivo animal testing methods have been used to characterize the sensitization potency of individual chemicals and their potential to specifically cause respiratory sensitization.

#### Guinea pig inhalation test

In this test, the respiratory sensitization capacity of individual chemicals is evaluated by determining the pulmonary responses to inhalation exposure in sensitized guinea pigs (Karol, 1994). Initial sensitization can be induced by inhalation or dermal exposure followed several weeks later by inhalation challenge (Karol et al., 1985; Pauluhn and Mohr, 2005; Sarlo and Ritz, 1997). Utility of this test derives from the fact that guinea pigs have challenge-induced pulmonary reactions similar to human asthma (Pauluhn and Mohr, 2005).

There are several important limitations to this test. Although the guinea pig response seems similar to clinical responses in humans, respiratory hypersensitivity in guinea pigs is primarily mediated by immunoglobu-lin *G_1_* (IgG1), whereas humans develop predominantly IgE-mediated hypersensitivity responses (Pauluhn and Mohr, 2005; Pretolani and Vargaftig, 1993). Also, guinea pigs “respond vigorously” to inhaled irritants, leading to asthma-like bronchospasm (Briatico-Vangosa et al., 1994; Kimber et al., 2007; Pauluhn and Mohr, 2005; Sarlo and Ritz, 1997). Because MMA and most other LMW respiratory sensitizers have irritant properties (Pauluhn and Mohr, 2005), distinguishing between sensitizer- and irritant-induced effects maybe difficult. Incorporation of a nonsensitized challenge group and use of smaller non-irritating exposure doses facilitate distinguishing irritants from sensitizers (Karol, 1994). Only a limited number of LMW chemicals have been tested and there are insufficient data to determine the test's sensitivity, specificity, and predictive value (Boverhof et al., 2008; Sarlo and Karol, 1994; Seed et al., 2008).

#### Mouse IgE test

This test measures IgE in BALB/c mice or Brown Norway rats following induction of sensitization. Exposure involves serial dermal applications followed 14 to 21 days later by blood sampling and measurement of IgE (Briatico-Vangosa et al., 1994; Karol, 1994; Pauluhn and Mohr, 2005). The presence of antigen-specific IgE or an increase in total IgE provides qualitative evidence of a compound's potential as a respiratory sensitizer. Although the test yields quantitative results, the magnitude of IgE response does not necessarily serve as a quantitative estimate of sensitizing potential (Boverhof et al., 2008; Briatico-Vangosa et al., 1994; Isola et al., 2008; Kimber et al., 2007; UN, 2007a).

#### In vivo cytokine profiling

In vivo cytokine profiling involves characterization of the cytokines produced by lymph node cells (LNCs) draining the areas where LMW sensitizers were applied (de Jong et al., 2009; Dearman et al., 2003a, 2003b). Respiratory sensitization is generally associated with secretion of anti-inflammatory Th2 cytokines, whereas contact sensitization and allergic contact dermatitis (ACD) are associated with inflammatory Thl cytokines. Respiratory irritation is not dependent on T-helper cells and irritant exposure does not normally lead to either of those cytokine patterns.

Cytokine profiling is most often performed in BALB/c mice sensitized with serial skin applications of test chemical. About 13 days after initial exposure, draining lymph nodes are excised and LNCs are cultured with and without mitogenic stimulation (e.g., concavalin A). A similar protocol has been adapted to inhalation exposure. Typically, mice are sensitized by inhalation on 3 consecutive days; 3 days later the draining lymph nodes are excised and treated as described above (de Jong et al., 2009). In both approaches, the culture supernatant is analyzed for cytokine protein by ELISA or cytokine bead array. An alternative approach involves measurement of cellular cytokine mRNA (Dearman et al., 1996, 2008; Hayashi et al., 2001; Kimber et al., 2007).

Despite its theoretical attraction, the practical value of cytokine profiling is uncertain. In some studies, expected cytokine patterns were not found after sensitizer exposures (de Jong et al., 2009; Dearman et al., 2003b; Selgrade et al., 2006; Vandebriel et al., 2000). In others, exposure led to co-expression of Thl and Th2 cytokines such that distinctions between contact and respiratory sensitizers were blurred (Arts and Kuper, 2007; de Jong et al., 2009; Ulrich et al., 2001). Moreover, discordance has been reported between the respiratory response predicted to occur on the basis of measured cytokine profiles and those actually observed (Pauluhn, 2008; Selgrade et al., 2006). Accordingly, cytokine profiling is viewed as a promising approach but one that requires additional work to optimize and validate testing protocols and test endpoints (Kimber et al., 2007; Roggen et al., 2008; Seed et al., 2008; Selgrade et al., 2006; Vandebriel and van Loveren, 2010).

#### Local lymph node assay

Local lymph node assay (LLNA) has been adopted as a stand-alone test by the National Toxicology Program (ICCVAM, 1999) and as the method of choice under REACH (OECD, 2002, 2009; van Loveren et al., 2008) for evaluation of contact skin sensitization. As described below, LLNA has also been performed following inhalation exposure to characterize the potential for respiratory sensitization. The test measures lymphocyte proliferation in lymph nodes draining the site where chemicals were applied. The method assumes that LNC proliferative responses are causally and quantitatively associated with the effectiveness of sensitization induction (ECETOC, 2003, 2008), yielding results that quantitatively describe relative sensitizing potency, which correlates with human sensitization thresholds (Loveless et al., 2010; van Loveren et al., 2008). Although initially used to evaluate induction of sensitization by skin exposure, recent studies describe its use for evaluating inhalation-induced sensitization (Arts et al., 2008; de Jong et al., 2009).

LLNA is usually performed in CBA or BALB/c mice. In a standard dermal LLNA, the chemical or a vehicle-only control is applied to the dorsum of the ears on 3 consecutive days. Three days later, the mice are injected with radiolabeled thymidine. After five hours, the draining auricular lymph nodes are excised and incorporation of radiolabel is measured by scintillation counting. At least three serial dilutions of the chemical are applied (Arts et al., 2008; de Jong et al., 2009). Results for test chemicals and vehicle controls are compared and expressed as a stimulation index (SI), calculated by dividing the scintillation counts for each chemical dilution or duration (per mouse or per lymph node) by the corresponding counts for the vehicle-only control. A chemical is considered “positive” and labeled a sensitizer if it induces an SI > 3 at any concentration or duration. However, some irritants (e.g., sodium lauryl sulfate) are reported to cause false positive LLNA (Basketter et al., 2009a; Roberts et al., 2007b).

The concentration or exposure duration corresponding to SI = 3 is referred to as the chemical's EC3, and that value has been used to classify sensitization potential (Basketter et al., 2000; ECETOC, 2008; Gerberick et al., 2007a; Griem et al., 2003; van Loveren et al., 2008). ECETOC defines four potency classifications on the basis of EC3 for chemicals tested by dermal exposure (Table 2) (ECETOC, 2008).

**Table 2:** Relative skin sensitization potency of contact allergens based on LLNA (ECETOC, 2008).

Potency rating	EC3 concentration (%)
Extreme	<0.1
Strong	≥0.1≤ to ≤1.0
Moderate	≥1.0≤ to ≤10
Weak	≥10

Although mainly used for dermal sensitizers, LLNA can also provide information relevant to respiratory sensitization. “Respiratory LLNA” has been performed using the inhalation exposures and measurement of lymphocyte proliferation in the mandibular lymph nodes draining the upper respiratory tract (Arts et al., 2008). For such testing, mice are exposed nose-only to the chemical or vehicle-only control over a range of exposure durations, e.g., 45-360 min/day for 3 days. Three days later, after injection of labeled thymidine, the mandibular lymph nodes are excised and treated as above (Arts et al., 2008; de Jong et al., 2009). Profiling of cytokines secreted by the draining lymph node cells after inhalation exposure has also been proposed as a basis for distinguishing between contact sensitizers, respiratory sensitizers, and irritants (de Jong et al., 2009).

The “inherent sensitizing potential of the chemical (potency)” is viewed as the most important factor external to the host for induction and elicitation of respiratory sensitization (Kimber et al., 2007) and EC3 has been proposed as the best approach for predicting sensitizing potency (Loveless et al., 2010). Almost all LMW respiratory sensitizers yield positive results on dermal LLNA, but most LMW chemicals with positive dermal LLNA are not respiratory sensitizers (Kimber et al., 2007). Thus, a negative LLNA implies that a tested chemical lacks respiratory sensitization potential (Arts et al., 2008; Boverhof et al., 2008; Kimber et al., 2007; Roggen et al., 2008).

#### MMA testing

*Guinea pig inhalation test.* Use of the guinea pig inhalation test has not been described for testing of MMA. *Mouse IgE test.* Use of the Mouse IgE Test has not been described for testing of MMA.

*In vivo cytokine profiling.* Use of in vivo cytokine profiling has not been described for testing of MMA. *Local lymph node assay.* Betts et al. (Betts, 2004; Betts et al., 2006) tested MMA using LLNA in CBA/Ca mice using two different solvent vehicles for skin application. In one test MMA was dissolved in pure acetone, and in the other MMA was dissolved in an acetone-olive oil mixture. MMA was weakly positive in both LLNA, with EC3 values of 90% and 60%, respectively. By contrast, a positive control contact sensitizer, 2,4-dichloronitrobenzene (DNCB), had an EC3 value of 0.036%, implying 3000-fold greater sensitizing potential. Based on LLNA results, the relative sensitizing potency of MMA can be compared to those of four acrylate monomers tested by the same researchers according to the same protocol (Dearman et al., 2007). As shown in Table 1, MMA is a substantially weaker sensitizer than most other methacrylates and acrylates.

In an early version of LLNA, Bull et al. (Bull et al., 1985) measured lymphocyte proliferation in the draining lymph nodes of guinea pigs after topical application of MMA dissolved in acetone-olive oil. Proliferation was scored by a microscopic cell-counting method. MMA exposure caused no increased proliferation. By contrast, methyl acrylate caused a significantly increased proliferation and was judged a “medium potential sensitizer."

### Contact skin sensitization

#### Background

Concerns that MMA might cause respiratory sensitization derive in part from its capacity to cause allergic contact dermatitis (ACD) in humans (US EPA, 1998; EU, 2002; IPCS, 1998) and numerous in vivo animal studies have been performed to characterize MMA skin sensitization. In addition to LLNA (described above), two other methods acceptable to US EPA and OECD (US EPA, 2003; OECD, 1992) for testing skin sensitization potential are the guinea pig maximization test (Magnusson and Kligman, 1970; Wahlberg and Boman, 1985) and the Buehler test (Buehler, 1965; Robinson etal., 1990). Under GHS (UN, 2007a), other “well-validated” methods such as the mouse ear swelling test (Gad et al., 1986) may also be used. Interpretation of test results is generally qualitative and criteria vary between methods and laboratories.

In addition, results vary across laboratories and depend on the route of exposure for induction of sensitization, vehicle used for skin applications, and the uses of adjuvants and occlusion (Basketter et al., 1993; Marzulli and Maguire, 1983).

#### MMA testing

The results of in vivo animal testing for MMA skin sensitization are presented in Table 3, which shows the test species, test methods for induction and elicitation of sensitization, the vehicle used for induction and elicitation, and the test results described by the individual authors. In some cases, only a very few animals were included in testing. A number of studies reported using multiple methods and varying vehicles, hence direct comparisons are difficult. In general, results indicate weak sensitization potential, although a significant proportion of studies had negative results. Negative results were more likely following topical applications with volatile vehicles (e.g., acetone, ethanol), probably because they allowed the MMA test material to evaporate thereby effectively reducing the applied dose.

**Table 3:** Summary of skin sensitization studies of MMA.

Species	Induction (sensitization) method	Elicitation (challenge) method	Solvent	Results	Reference
***Guinea pig maximization test***					
Guinea pig	Intradermal injections (day 0); occlusion (day 7)	Occlusion (day 21), open application (day 35)	Ind—80% ethanol;, peanut oil, or Aramek Elicit—Peanut oil or aramek	2/10 3/10	(118)
Guinea pig	Intradermal injections (day 0)	Ost (day 14, repeated up to 12 weeks)	Ind—Ethanol, FCA, saline Elicit—Acetone:olive oil (4:1)	0/6	(119)
Guinea pig	Intradermal injections (3 groups), occlusion (1 group)	Day 21 max nonnonirritating conc. (all groups), 24 24-h occlusion	Ind—Propylene glycol; FCA/propylene glycol; FCA; petrolatum Elicit—Non-Nonirritating conc Occlusion	0/5 2/5 2/5 4/5	(120)
Guinea pig	Intradermal injections (3 rounds, all day 0)	Day 21, 24-h occlusion	Ind—Group 1: FCA:distilled water (day 0) Group 2: MMA:saline (day 0) Group 3: MMA/saline:FCA (1:1) (day 0) All groups: 24-h occlusion (day 7) Elicit—Ethanol	0/10 0/10 0/10	(116)
Guinea pig	Occlusion (days 0, day 7)	Occlusion	Ind—(Day 0,, FCA, saline); (day 7, neat MMA) Elicit—Dilute MMA; 1% MMA: Vaseline,; 5% MMA:Vaseline (day, not described)	Negative (10% of animals treated with 1% conc.) Positive (50% of animals treated with 5% conc.)	{(121),(122)
Guinea pig	Intradermal injections (day 0) OST (day 7) Occlusion, 48 hour (day 8)	Occlusion (day 21)	Ind—Day 0, (FCA:Water, MMA: soybean oil, MMA:FCA:Water;) day 7, (MMA:Petrolatum, SDS); day 8, (neat MMA) Elicit—MMA 3%:petrolatum (occlusion)	9/10	(123)
Guinea pig	Intradermal injections (day 0), occlusion (day 7)	24 hr Occlusion, 24 h (day 21)	Ind—Group 1: (Day 0, 5% MMA:Water, 10% MMA:FCA.; day 7, neat MMA.; day 24, 5% MMA) Group 2: (Day 0, 0.15% MMA:water, 0.3% MMA:FCA.; day 7, 0.15% MMA.; day 24, 100% MMA) Group 3: (Day 0, 0.001% MMA:water, 0.002% MMA:FCA.; day 7, 0.001% MMA.; day 24, 100% MMA) Group 4: (Day 0, 0.5% MMA:water, 10% MMA:FCA.; day 7, 100% MMA.; day 24, 100% MMA) Elicit—Group 1: (5% MMA occlusion, 48 h) Groups 2–4: (100% MMA, 5% MMA, acrylic particles occlusion, 48 h)	Group 1 (4/26) Group 2 (0/13) Group 3 (0/12) Group 4 (20/26)	(124)
Guinea pig	Intradermal injections (day 0), occlusion (day 7)	Occlusion, 24 hr (day 14)	Ind—FCA, MMA:dibutylphthalate, MMA:FCA, acetone. Elicit—Acetone	0/10	(125)
Guinea pig	Intradermal injections and topical (day 0)	Occlusion, 24 hr (day 21)	Ind—Olive oil Elicit—Acetone	2/14 (1 M conc.) 0/10 (10^–1^ M conc.) 0/10 (10^–2^ M conc.)	(126)
Guinea pig	Intradermal injections Day 0: six 6 injections, six 6 sites, FCA.Day 7: SLS (24 hr)Day 8: occlusion (48 hr)	Day 21, occlusion (24 hr)	Ind—Olive oil:acetone (7:3) Elicit—neat MMA	6/6	(127)
Guinea pig	Intradermal injections, 3 control groups and 3 test groups, six 6 injections (day 0), SLS (day 6), occlusion (day 7, 48 hr)	Occlusion (days 20, Day 27; 28 hr, 48 hr, 72 hr)	Ind (test groups)—FCA:water (1:1), MMA:FCA (1%, 3%, 10% MMA)	Challenge (day 20): 1/4 (after 24, 48, 72 hr) 2/5 (after 24, 48, 72 hr) 5/5 (after 24, 48, 72 hr) Rechallenge: 1/4 (after 24, 48, 72 hr) 1/5 (after 24, 72 hr and 0/5 after 48 hr) 5/5 (after 24, 72 hr and 3/5 after 48 hr)	(128)
Guinea pig	Intradermal injections Day 0: six 6 injectionsDay 7: occlusion (Day 7, 48 hr)	Occlusion (day 14, 24 hr); OST (day 28)	Ind—FCA:Water (1:1), MMA 10%:maize oil, MMA 10%:FCA/water (1:1) Elicit —25% MMA; maize oil or maize oil/DMSO (day 14) then occlusion (24 hr), OST with 25 and 50% MMA in ethanol/40% DMSO (day 28).	5/5 5/5 5/5 5/5	(129)
***Beuhler***					
Guinea pig	Method A: OST (days 0, 2, 4, 7, 9, 11) Method B: OST (days 0, 1, 2, 3, 4, 7, 8, 9, 10, 11)	Method A: OST shaved flank (day 28, repeated up to 12 weeks)Method B: OST shaved neck (day 21, repeated up to 12 weeks)	Method A: Ethanol 95%:2-methoxyethaol: Tween 80 (9:9:2) Method B: Acetone: olive oil (1:1)	Method A: 0/6 Method B: 0/6	(119)
Guinea pig	OST, 6- hour occlusion (days 0, 7, 14)	24- hour occlusion, day 28	Ind—neat MMA Elicit —Ethanol	0/30	(116)
***Freund's complete adjuvant test (FCAT)***					
Guinea pig	Intradermal injections in shoulder (days 0, 2, 4, 7, 9)	Occlusion (day 21),); OST (Day 35)	Ind—FCA Elicit—Peanut oil or Aramek (occlusion day 21); peanut oil or Aramek (day 35, OST)	2/8	(118)
Guinea pig	Intradermal injections multi-site (day 0)	Occlusion (day 14, 24 hr); OST (day 28)	Ind—1 M MMA:FCA/saline Elicit—2.5 M in maize oil (occlusion); 2.5 M in DMSO/maize oil (occlusion); 2.5 M in ethanol (OST); 5 M in ethanol (OST)	5/55/54/55/5	(129)*
***Split adjuvant test***					
Guinea pig	Multi-Site intradermal injections of dorsal shaved flank (day 0: FCA, day 1: MMA)	OST (day 14, repeated up to 12 weeks)	Ind—Ethanol:saline (1:100) Elicit—Acetone:olive oil (4:1)	0/6	(119)
Guinea pig	Frozen (dry Ice contact, day 0), occlusion (days 0, 3, 4, 7), intradermal injections (day 4)	Day 22, 24-h occlusion	Ind—Dry ice, ethanol, FCA:ethanol:MMA, ethanol Elicit—Ethanol	0/30	(116)
***Polak***					
Guinea pig	Footpad injections and intradermal injections	OST (day 7, repeated up to 12 weeks)	Ind—Ethanol:saline (1:4) Elicit—Acetone:olive oil (4:1)	0/6	(119)
Guinea pig	Footpad injections and intradermal injections (nape of neck)	Group 1: OST (day 0, reactions recorded days 2, 4, 5, 6) Group 2: OST (crossreactivity) days 7, Day 14	Ind—Ethanol:saline (1:4) in FCA occluded, some also injected. Elicit—Acetone: olive oil (4:1)	0/5 (8 groups)	(130)
Guinea pig	6 groups: 2 hind footpad injections each group (day 0),); group 1 occlusion day 0), groups 2–3 occlusion (day 10), groups 4–5 occlusion (day 25), group 6 intradermal injection (day 25).	Groups 1–3, OST (day 35).); groups 4-6, OST (day 60)	Ind—FCA (groups 1–6) olive oil (groups 1, 3, 5); ethanol (groups 2, 4); saline injection (group 6) Elicit—Olive oil (groups 1–3, day 35); olive oil (groups 4–6, day 60)	Group 1 (0/10) Group 2 (0/5) Group 3 (0/5) Group 4 (0/5) Group 5 (0/5) Group 6 (0/5)	(131)
Guinea pig	Part A:6 groups: Footpad injections in each group (day 0).); OST, (all groups, days 0, 2, 5); group 4, additional intradermal injection (day 0); group 5, OST (day 25); group 6 intradermal injection (day 25).Part B:Group 4, (same as above);group 7, (footpad injection (day 0), intradermal injection (day 0)	OST (groups 1–3, day 35), OST (groups 4–6, day 60)	Ind—FCA (all groups), ethanol (groups 1–5), saline (groups 4 and 6) Elicit—Ethanol (groups 1–4), olive oil (groups 5 and 6)	Part A: Group 1 (0/25, 72 hr) Group 2 (0/24, 72 hr) Group 3 (0/25, 72 hr) Group 4 (0/10, 72 hr) Group 5 (13/13, 72 hr) Group 6 (8/8, 72 hr) Part B: Group 4 (1/10, 48 hr) Group 7 (2/10, 48 hr)	(132)
***Draize***					
Guinea pig	10 intradermal injections over 1 week.	Intradermal injection 14 days after last induction injection (24-hour determination)	Ind—Saline Elicit—Ethanol	2/30	(116)
***Mouse ear sensitization test (MEST)***					
Mouse	Intradermal injection (day 0), topical application (days 0–3)	Topical application to ear (day 10)	Ind—FCA, Ethanol Elicit—Ethanol	44% sensitized, 118% increase in ear thickness	(115)
Mouse	Topical application (days 0, 2)	Topical application (day 9)	Ind—Ethanol Elicit—Ethanol	114% increase in ear thickness (24 hours after challenge, *p* < 0.01)	(133)

*Note*. Ind = vehicle used for the “Induction (Sensitization)” procedure.;

Elicit = vehicle used for the “Elicitation (Challenge)” procedure.;

FCA = Freund's complete adjuvant;

OST = open skin testing;

SLS = sodium lauryl sulfate;

SDS = sodium dodecyl sulfate.

* = Modified FCAT method.

## Human studies

### Background

Numerous human studies have considered the adverse respiratory health effects of MMA exposure, although many did not specifically address the distinction between respiratory irritation and respiratory sensitization. As detailed below, studies range from simple surveys of self-reported complaints to descriptions of sophisticated physiological and diagnostic testing. The accumulated literature includes epidemiological studies *(cohort studies, cross-sectional studies,* and *mortality studies),* and *case reports* of occupational asthma, occupational rhinitis and laryngitis, and hypersensitivity pneumonitis.

Many of the studies suffer from informational deficits. As described below, studies rarely confirmed self-reported complaints and diagnoses, whereas most relied on clinical and diagnostic tests that have only limited predictive value for asthma. Thus the clinical status of most of the workers described in the epidemiology studies is uncertain.

The limited adequacy of work site exposure assessments reported in these studies is another important source of uncertainty. For example, as described in epidemiological studies below, most workers’ exposures were not measured on most days and exposure levels were generally described in terms of relatively broad ranges. Also, workplace exposures were almost always reported as long-term (i.e., full-day) time-weighted averages; short-term peak exposures were rarely identified or measured. The importance of this is seen in the case reports, which mainly describe workers likely to have suffered short-term peak exposures.

In addition, accurate measurement of airborne MMA is subject to technical challenges that contribute to underestimation of exposure levels and that have often been ignored. Finally, the exposure of most workers was described for MMA, but many of those workers were also exposed to airborne mixtures, including cross-reacting compounds and other respiratory sensitizers and irritants. The following two sections consider the technical challenges for MMA measurements and the likely significance of mixtures.

#### Exposure measurements: Technical challenges

Recently, concerns have been raised that the methods used for assessing exposures reported in epidemiologi-cal studies and case reports may significantly understate actual exposure levels (Ungers et al., 2007; Ungers and Vendrely, 2006). One concern is the propensity of MMA to rapidly polymerize on sampling media, thus forming unrecoverable polymer. Most historical sampling methods did not utilize inhibitors or store samples on dry ice, as is currently recommended (NIOSH, 2003; OSHA, 2010). Another concern is the relative insensitivity of analytical methods; deviations from standard sampling and analytical protocols (e.g., NIOSH, 2003; OSHA, 2010) were often required to detect and quantify short-term (e.g., 15-minute) exposures. For example, higher than recommended air flow rates, used in order to achieve lower than standard levels of detection, can result in sample breakthrough, MMA loss, and underestimation of exposure.

**Figure 2:**
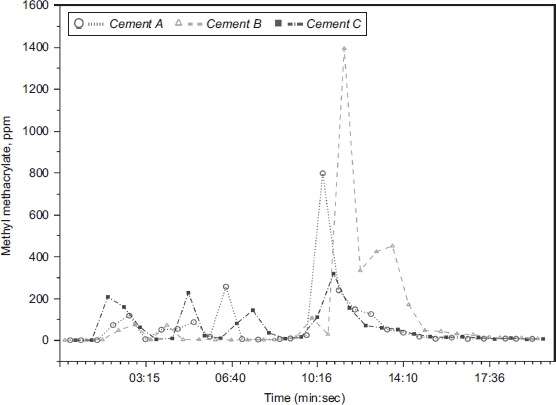
MMA air levels measured above a mixing bowl during the mixing of MMA-containing orthopedic cement. Measurements were obtained every 45 seconds over 20 minutes using a photo-acoustic spectrophotometer. Cement A = Endurance® (DePuy); Cement B = Palacos-R® (Schering-Plough); Cement C = Simplex-P® (Stryker). (previously unpublished data courtesy of Leslie J. Ungers, MS, CIH; see also Ungers et al., (2007); Ungers & and Vendrely, (2006)). (See colour version of this figure online at www.informahealthcare.com/txc)

Such exposure assessment concerns have special importance for the interpretation of specific inhalation challenge (SIC) testing as described in case reports. SIC was performed as workplace simulations for 20 (Lozewicz etal., 1985) or 30 (Savonius etal., 1993) minutes, but actual exposure levels were described in only one report. In that study, MMA exposure was monitored with an infrared gas analyzer; MMA levels reached 374 ppm 2 minutes after Palacos® R bone cement was mixed (Pickering et al., 1986), but levels were not described over the duration of the test. More recent studies have found that MMA levels continue to increase for 10-20 minutes after mixingbone cement, reaching levels > 1000 ppm above the mixing bowel if emission controls are not employed. Such high peak levels are illustrated in Figure 2, which presents previously unpublished data from studies by Ungers and colleagues (Ungers and Vendrely, 2006), who performed workplace simulations using three different MMA-containing bone cements. For each cement, samples were obtained about every 45 seconds over 20 minutes using a photo-acoustic spectrophotometer. Thus, SIC workplace simulations may expose SIC test subjects to MMA peak levels nearly 15 times current occupational exposure limits.

#### Mixtures and cross-reactivity

The epidemiological studies and case reports described below are limited by uncertainties regarding the exposures of concern. This is of particular importance for those case reports that performed specific inhalation challenge (SIC) without independently determining the composition of the test material. The problem is that methacrylate-containing materials are almost always mixtures of methacrylates and acrylates, which contain inhibitors, activators, and other reactive compounds that are known sensitizers and irritants.

These concerns have been best documented for dental materials. Vankerckhoven et al. (Vankerckhoven et al., 1981) used HPLC, GC, and NMR to characterize the composition of dental resins and bonding agents and detected numerous non-MMA methacrylates. Rustemeyer and Frosch determined that dental product Material Safety Data Sheet (MSDS) often lacked details about the various acrylates, methacrylates, and additives included in dental materials, often because manufacturers viewed such details as trade secrets (Rustemeyer and Frosch, 1996). In studies at the Finnish Institute of Occupational Health, Kanerva and colleagues used GC-MS to analyze the composition of acrylate-containing dental products and to determine acrylate air levels in dental clinics. Most analyzed products contained acrylates and methacrylates known to be sensitizers that were not declared on MSDS (Henriks-Eckerman and Kanerva, 1997).

The methacrylates found most often in dental resins and b onding materials were 2 -hydroxyethylmethacrylate (2-HEMA), 2,2-bis[4-2-hydroxy-3-methacrylopropoxy) phenyl]propane (bis-GMA), and triethyleneglycol dimethacrylate (TREGDMA) (Henriks-Eckerman et al., 2004). Kanerva et al. (Kanerva et al., 1994; Kanerva et al., 1997) reported that TREGDMA and ethyleneglycol dimethacrylate comprised up to 37% of product weight in some dental preparations. Yoshii (Yoshii, 1997) evaluated “thirty-nine acrylates and methacrylates that had been used in dental resin materials,” but the basis for his identifying them was not described. Another perspective is provided by air monitoring studies in six dental clinics that utilized high sampling rates (300 ml/min) to achieve low detection limits (0.5 μg/m^3^). All six clinics had measurable levels of 2-HEMA and TREGDMA, but MMA was “not detected” in all of the clinics (Henriks-Eckerman et al., 2001).

Dental materials also contain nonacrylate additives that are often not listed on MSDS and product labels. Examples include reaction initiators (e.g., benzoyl peroxide), reaction activators (e.g., tertiary amines such as N,N-dimethyl-p-toluidine 4-tolyl diethanolamine), cross-linking agents (e.g., formaldehyde), reaction inhibitors (e.g., hydroquinone and p-methoxyphenol), and resin carriers (e.g., N-ethyl-4-toluene sulfonamide) (Kanerva et al., 1989, 1997; Van Der Walle et al., 1982a). All of these additives are known contact sensitizers and several are known or suspected respiratory sensitizers and/or respiratory irritants (Enoch et al., 2009; Geurtsen, 2000; Kanerva et al., 1997; Van Der Walle et al., 1982a). In addition, contaminants and impurities (e.g., diglyci-dyl ether of bisphenol A) have been associated in case reports with respiratory sensitization and OA (Kanerva etal., 1991, 2000).

In some studies, researchers failed to list all known sensitizing components in the materials of concern. For example, responding to inquiries by an ECETOC task force about the cases reported in dental workers (Savonius et al., 1993;), the authors acknowledged that “workers had been exposed to other acrylates than methyl methacrylate,” that the material used in SIC testing was “impure,” and that subsequent analysis determined that dental materials “contain many additional acrylates that have not been decleared [sic] in the MSDS... additives and additional impurities” (Kanerva, 1993).

Similar mixture issues pertain to bone cements and cosmetic products, but they have been less systematically studied (Thomas et al., 2008). Granchi et al. described differing compositions of 10 bone cements, but they relied on manufacturers’ declarations rather than independently analyzing the materials (Granchi et al., 2002). All 10 contained benzoyl peroxide and N,N-dimethyl-p-toluidine, whereas 9 contained hydro-quinone. Some contained acrylates other than MMA, such as butyl methacrylate (BMA) and methyl acrylate. In addition, some bone cements contain antibiotics at levels up to 10% by weight ("antibiotic-loaded cement") (Jiranek, 2005; Jiraneket al., 2006). For example, Palacos® R and CMW® contain gentamicin and Simplex® P contains tobramycin (Connolly et al., 2007; Schulze and Wollina, 2003), antibiotics that cause IgE-mediated allergic reactions (Connolly et al., 2007; Hall, 1977; Nikolaizik et al., 2002; Schretlen-Doherty and Troutman, 1995; Schulze and Wollina, 2003). Likewise, GC-MS analysis of the resins and bonding agents used by an affected cosmetic nail stylist identified 11 different methacrylates (Sauni et al., 2008). The two most abundant were HEMA (8%) and bis-GMA (42%), whereas MMA represented <0.05%.

Because most test compounds were mixtures of known sensitizers, the results of SIC using those compounds can indicate that patients suffered OA or occupational rhinitis, but they cannot identify the causal agent in the mixture. A further complication is that many of the agents cross-react, raising uncertainty about which agent was responsible for induction of hypersen-sitivity. Such cross-reactivity has been demonstrated in animals and humans using skin test protocols. In guinea pigs, cross-sensitization has been shown between MMA and the following acrylates and methacrylates: ethyl methacrylate (EMA) and BMA (Chung and Giles, 1977); 2-HEMA, 2-hydroxypropyl methacrylate (2-HPMA), and ethyleneglycol dimethacrylate (EGDMA) (Rustemeyer et al., 1998); and, EMA, BMA, ethyl acrylate, n-butyl acrylate, and neopentyl acrylate (Van Der Walle and Bensink, 1982). In addition, concomitant sensitization to reaction inhibitors (hydroquinone and *p*-methoxy-phenol) occurred with 7 of 11 methacrylates reported to be otherwise >99% pure; levels of those inhibitors in the methacrylate mixtures were not reported (Van Der Walle et al., 1982a). Based on frequency of positive tests and capacity to induce broad cross-reactivity in guinea pigs, Rustemeyer et al. judged 2-HEMA to be the “the most clinically relevant methacrylate contact sensitizer” (Rustemeyer et al., 1998).

Human patch testing with methacrylates have demonstrated apparent cross-reactivity. Goon et al. reported patch test results in 1632 patients, of whom 48 had positive results to one or more methacrylates (Goon et al., 2006). Positive responses were most frequent for 2-HEMA (47/48, 98%). Of those positive for 2-HEMA, responses were also noted to EGDMA in 63% (30/47), TREGDMA in 27% (13/47), and MMA in 23% (11/47). Of the 11 patients who responded to MMA, all responded to 2-HEMA, 10 responded to EGDMA, 9 responded to TREGDMA, and 6 responded to 1,4-butanediol dimethacrylate. Rustemeyer and Frosch (Rustemeyer and Frosch, 1996) performed patch testing in 35 dental technicians with allergic contact dermatitis and found that multiple methacrylate sensitizations were common. Positive results were most frequent for 2-HEMA (18/35, 51%), EGDMA (15/35, 43%), and MMA (9/35, 26%). Of 16 patients with multiple sensitivity, all responded to 2-HEMA, 14 responded to EGDMA, and 7 responded to MMA. Roche et al. (Roche et al., 2008) studied 15 women with contact sensitization caused by acrylic fingernails. All 15 patients responded to at least two, and in some cases up to eight, acrylates and methacrylates.

### Epidemiological studies

The epidemiological studies summarized below aimed to determine whether MMA-exposed workers hadincreased rates of respiratory symptoms, respiratory dysfunction, or histories of upper and lower respiratory tract diseases, e.g., rhinitis, bronchitis, and asthma. Most did not verify self-reported diagnoses and, as described below, none could determine whether noted effects were specifically due to sensitization or irritation. Moreover, as discussed earlier, it can be clinically difficult to determine that an individual suffers sensitizer- versus irritant-induced respiratory disease.

There are several other sources of uncertainty in these studies. One source is the inherent limitation of questionnaire information, such as subjective symptoms and self-reported diagnoses, which are generally not amenable to validation. None of the studies described below included reviews of medical records or external evidence of MMA-related disease. Another source of uncertainty is the limited adequacy of work site exposure assessments. Most industrial workers are exposed to mixtures, but reported exposure assessments generally considered only MMA. In addition, the actual exposures of most workers were not measured on most days, thus exposure levels were described in terms of relatively broad ranges; short-term peak exposures were rarely identified.

A third source of uncertainty derives from the diagnostic tests commonly used in work site screening for respiratory diseases such as asthma. Tests such as spirometry and peak expiratory flow rates (PEFRs) have only limited predictive value for asthma, especially when performed in nonstandard ways (Bernstein et al., 2006a; Burge et al., 2006; Nicholson et al., 2005; Tarlo et al., 2008). For example, a single spirometry determination of FEV1 (forced expiratory volume in one second) is recognized to have very limited diagnostic value, whereas cross-shift spirometry is “insensitive” (Balmes, 1991; Bernstein et al., 2006a; Burge, 1982; Nicholson et al., 2005). PEFR has only somewhat greater sensitivity and specificity than serial spirometry. A recent meta-analysis that evaluated PEFR versus specific inhalation challenge (SIC) reported pooled sensitivity of only 56.2% (95% confidence interval [CI]: 17.2-88.8%) and pooled specificity of only 77.2% (95% CI: 66.5-85.2%) (Beach et al., 2005). Moreover, PEFR results depend on a patient's effort and integrity because testing is usually unsupervised (Hankinson, 2000; Leroyer et al., 1998; Perrin et al., 1992).

#### Cohort studies

*Acrylic sheet manufacture.* Pausch et al. (Pausch et al., 1994) conducted semiannual examinations over a 2-year period (1991-1993) in 211 male German acrylic sheet workers (mean age 37 years) engaged in the production of acrylic sheets for an average of 8.8 years (range: <1 to >20). Personal air samples for MMA were used to estimate current exposure levels in four different work areas (8-hour time-weighted averages [TWAs] of 3-10, 10-20, 20-30, and 30-40 ppm); however, historical exposures were noted to have been “considerably higher.” Short-term peak concentrations (100-680 ppm) were observed on occasion in all areas. Exclusion/inclusion criteria were not reported. A group of 55 recently hired workers (mean age 32 years) without previous exposure to MMA was characterized as a comparison group, but their outcomes were not reported.

Examination included a self-administered questionnaire that emphasized complaints related to nose and throat, but also included questions about the respiratory system, “asthmatic reactions,” and allergic skin reactions. Physical examination was limited to speculum-guided anterior rhinoscopy. Symptoms were considered unrelated to MMA if nonwork causes could be identified (e.g., hay fever, deviated nasal septum) if symptoms existed prior to MMA exposure, or if symptoms occurred at home. Among workers with the highest exposures (30-40 ppm; *n = 56),* none of the reported nasal symptoms (e.g., “impaired nose breathing,” “dry nose,” and “rhinitis") were related to MMA exposure. Among workers in lower exposure categories, the prevalence of MMA-related nasal symptoms ranged from 1% to 10%; effects were described as transient and associated with high-peak exposures (100-680 ppm of 5-15 minutes duration).

A “reduced sense of smell” was reported by 4 workers (2/128 at 10-20 ppm and 2/20 at 20-30 ppm); 2 of those workers (1 case from each exposure group) were active smokers. MMA-related “eye irritation” (burning, itching, or lacrimation) was reported in 3 workers (2/128 at 10-20 ppm and 1/20 at 20-30 ppm). Reports of “chronic bronchitis” were limited to 2 workers with low-level MMA exposures (1/7 at 3-10ppm and 1/128 at 10-20ppm) and exposure durations of 3 and 36 years. There were no reports of skin or respiratory sensitization associated with MMA exposure and examination found no clinical evidence of effects on the nasal epithelium.

This report provided evidence of reversible irritation to the eyes, nose, and upper respiratory tract associated with short-term MMA peaks > 100 ppm, but no evidence for respiratory sensitization in workers with long-term moderate to high-level MMA exposures. The strength of these findings is limited by their subjective nature (self-reported “asthmatic reactions"). Because all workers were rotated through different areas, and potentially exposed to high peaks, it is also difficult to draw conclusions regarding the role of exposure levels and dose.

#### Cross-sectional studies

*Acrylic sheet manufacture.* Cromer (Cromer, 1976) conducted a NIOSH study (not peer reviewed) that involved the screening of 91 male volunteer workers for potential health effects associated with MMA exposure during acrylic sheet production at five different US plants. Workers at one plant were also exposed to ethyl acrylate (8-hour TWA of 0.5-2.0 ppm). Duration of employment was not reported. Inclusion and exclusion criteria were not defined, but two workers with previous welding exposure were excluded. Personal sampling data were used to calculate mean 8-hour TWA exposures by job category and workers were divided into four exposure groups: Group 1: <5ppm or 2 months duration; *n=13,* mean age 41.5 years, 39% smokers; Group 2: 5-25 ppm; *n = 20,* mean age 40.3 years, 45% smokers; Group 3: 25-50ppm; *n = 33,* mean age 35.3 years, 70% smokers; Group 4: “not currently exposed,” but previous exposure > 1 year; *n =* 25, mean age 51 years, 48% smokers. Group outcomes were compared to findings in 43 nonexposed control workers (mean age 45 years) employed by the same company, but worked in different buildings. “Controls” were significantly older than Group 3 workers, (p < .001), significantly younger than Group 4 workers (p= .025), and had significantly lower prevalence of smokers than Group 2 workers (p<.04).

Workers were given a self-administered questionnaire regarding work history, and medical history and respiratory symptoms (cough, phlegm, breathlessness, wheezing); symptom prevalence and exposed versus control group differences were reported for only selected symptoms. In currently exposed workers, the prevalence of “cough” decreased with increasing exposure (23% at <5ppm, 20% at 5-25 ppm, and 15% at 25-50 ppm), but it was lower in workers “not currently exposed” (12%) and lowest in controls (7%). Significant differences were only observed between the lowest exposed workers (23% at <5ppm) versus controls (7%) (p <.O3). Prevalence of sputum production was not dose related, ranging from 23% at <5ppm, to 50% at 5-25 ppm, to 21% at 25-50 ppm in currently exposed groups, 32% in “not currently exposed,” and 14% in controls. The highest prevalence of cough and sputum production was noted in the groups that had the highest prevalence of smokers, 62% in the lowest exposure group and 70% in the medium exposure group. Outcomes for symptoms of breathlessness and wheezing were not reported. No significant differences in the self-reported prevalence of skin or allergic problems were noted between the exposed and control groups.

Spirometry was performed at one time point and findings were compared to a normative data base (Kory et al., 1961; Lapp et al., 1974). Differences between exposed and control groups in predicted-minus-observed values were evaluated using Student's *t* test; outcomes for smokers and nonsmokers were analyzed separately. There were no significant differences in spirometry results. Among the exposed workers, spirometry results were better than predicted except for MMF, and were the same or better than the results for control workers. There were no significant differences in spirometry when nonsmoking exposed workers were compared to nonsmoking controls.

Findings of this study are limited by the performance of only a single spirometry and the small size of comparison groups.

Monroe (1981), in an unpublished report, described findings of a respiratory health survey of 780 employees at a US Plexiglas® manufacturing facility. The study was undertaken because earlier clinical observations had suggested an increased prevalence of pulmonary disease. The study group represented 94.4% of eligible workers; reasons for nonparticipation included vacation, absence from work, and missed appointments. MMA exposures varied across job functions and over time. Before 1976, TWA and short-term exposure levels (STELs) ranged up to 89.9 and 134 ppm, respectively. By 1979, TWA levels were <50ppm, but STELs up to 125 ppm were observed in two operations. Workers were also exposed to two other respiratory irritants, ethyl acrylate and formaldehyde, but at levels below their respective occupational exposure limits.

Evaluations included a self-administered respiratory questionnaire, chest X-ray, and spirometry performed once according to 1978 American Thoracic Society (ATS) standards (ATS, 1979). Workers were divided into three groups: Group 1: MMA-exposed workers (*n* = 68, mean age 38.6 years, work duration 12.1 years); Group 2: mechanics (*n* = 80, mean age 47.4 years, work duration 22.3 years); and Group 3: control group consisting of all other employees (*n* = 632, mean age 43.5 years, work duration 17.3 years). The study considered gender, work duration, and smoking history (current, ex-, “other” pipe/cigar smokers, and never) and intensity (number of cigarettes/day, lifetime cigarette consumption).

Spirometry results were compared to a “normal population” (Morris et al., 1971) and across groups. None of the MMA-exposed workers had “abnormal” spirometry ("abnormal” defined as <70% of predicted). Group 1 did not differ from the other groups, except that the never-smoking MMA-exposed workers had significantly lower FEV1 than never-smoking control workers (*p* <.005). The prevalence of less-than-predicted spirometry results was greater among current-smokers than other smokers and never-smokers(*p* < .025). Chronic bronchitis was reported by 3 of 68 MMA-exposed workers (4%), 87 of 632 control workers (14%), and 12 of 80 mechanics (15%); prevalence was significantly lower in Group 1 (*p* <.O26).

The significance of these study findings is limited by the use of only a single spirometry and the fact that the MMA-exposed workers included significantly fewer and less intensive smokers than the mechanics and control workers.

Della Torre et al. (Della Torre et al., 1982) investigated the potential health effects of MMA in 18 Italian workers involved in the manufacture of Plexiglas® sheets. Job-exposure data divided workers into two groups based on mean exposure levels: Group A was exposed to >100ppm and Group B was exposed to <100ppm MMA. Concentrations of MMA ranged from 115.4 to 180 ppm in Group A (*n* = 8, 4 females, 4 males; mean age 35.2 years, duration of exposure 14 years) and 4.8-83.5 ppm in Group B (*n* = 10, 4 females, 6 males; mean age 36.6 years, duration of exposure 10 years).

The prevalence of self-reported symptoms of eye and throat irritation was assessed by questionnaire and compared to clinical examination findings. The number of workers who complained of “eye irritation” (Group A: 87.5%, Group B: 20%) greatly exceeded the number of workers with clinical evidence of eye irritation (Group A: 37.5%, Group B: 10%). Similarly, symptoms of “throat irritation” were reported by 62.5% of Group A workers, but clinical signs of throat irritation were observed in only 37.5%.

The prevalence of *episodic* “productive cough” was greater in Group A (50%) compared to Group B (20%), as was prevalence of *frequent* “productive cough” (Group A: 25%, Group B: 10%). Three workers had been previously diagnosed with chronic bronchitis: one had also undergone laryngectomy (details not provided), a second was a heavy smoker, and the third had been exposed to combustion products during maintenance of a boiler. Spirometry revealed no functional deficits in any of the workers; details were not reported.

The findings of this study are limited by the lack of a concurrent nonexposed comparison group and the small sample size.

Mizunuma et al. (Mizunuma et al., 1993) investigated potential methods for biological monitoring of MMA exposure and also briefly reported on the prevalence of subjective complaints in 32 Japanese workers (mean age: 43.3 years, range 21-60 years) exposed to MMA during the production of MMA polymer (pMMA) sheets. Average exposure levels, determined from personal samplers (carbon cloth) worn at chest level for full 8-hour shifts, ranged from 0.4 to 112.3 ppm (geometric mean [geometric standard deviation], GM [GSD]: 6.1 ppm [4.5]); 4 workers experienced levels >50ppm, one of which exceeded 100 ppm. Workers were divided into two exposure subgroups: “high” (median 18 ppm, range 5-112ppm, *n=16)* and “low” (median 1ppm; range <5ppm, *n=* 16); duration of exposure was not reported. Results were compared to a group of 16 apparently non-exposed clerical workers (mean age: 37.8 years, range 22-60 years).

MMA-exposed workers reported a significantly greater prevalence of “frequent cough and sputa” (6/32, 19%) and “throat irritation” (4/32, 13%); none of the controls complained of such symptoms. Symptom prevalence appeared dose related: four of six cases of “frequent cough and sputa” and all four workers reporting “throat irritation” were from the high-exposure group.

This study failed to consider the effects of smoking and exposures to other workplace irritants; duration of exposure was not described; there was no attempt to validate the subjective complaints; and the control group was of uncertain comparability to the exposed workers.

Pickering et al. (Pickering et al., 1993) investigated the prevalence of occupational asthma attributable to MMA exposure in 384 of a total 431 workers (380 male; mean age: 37 years) employed in three English acrylic sheet-producing factories. Reasons for nonparticipation included refusals, vacations, and “unavailable.” Individual exposures were estimated using a job-exposure matrix and industrial hygiene data that included area, but not personal samples. Based on estimated 8-hour TWA exposures, workers were categorized into three groups: low-exposure ("<1 ppm"; *n=157),* medium-exposure ("5 ppm"; *n =* 163), and high-exposure ("20 ppm"; *n =* 64). The authors did not explain whether these exposure levels represented means, medians, or upper bounds. Doubts about the exposure scheme were raised in a Health and Safety Executive (HSE) criteria document, which reported that the “arithmetic mean exposure was approximately 22 ppm” and that “a significant proportion of workers would have been exposed to average concentrations of approximately 50 ppm” (Cary, 1995).

Examinations included an administered respiratory questionnaire and spirometry. Spirometry readings were performed in 380/384 and findings were compared to a normative database. Symptoms (persistent cough, chronic bronchitis, chest tightness, wheeze, and breath-lessness) were considered work related if they were reported to be exacerbated during a work shift, or if they improved over weekends and holidays.

The prevalence of work-related symptoms was low (1.0-3.4%, 7-13 individuals for each symptom), particularly when compared to the prevalence of non-work-related symptoms (4.2-9.9%; 16-38 individuals). There was a significant excess of smokers (current and past) among workers who reported work-related symptoms (25 of 27; 92.6%) compared to the proportion of smokers in the total study population (255 of 384; 66.4%). Nine workers (2.3%) reported two or more respiratory symptoms, but none gave a history of work-relatedness consistent with OA. Transient high exposures to MMA were reported by 70% of workers and were associated with increased frequency of cough, chest tightness, shortness of breath (SOB), wheezing, and eye and nasal irritation. The prevalence of symptoms related to transient high exposures was significantly greater among ever-smokers than never-smokers (*p* <.O5). Symptoms were significantly reduced among workers who had never experienced transient high exposures compared to those that had (*p* <.O5). Stepwise forward logistic regression identified “past or present smoking history” as a significant predictor of work-related respiratory symptoms (*p* <.01), whereas the “frequency of high exposure incidents” was of borderline significance (p=.O5).

Spirometry results were compared to predicted normative values, not to a control group. In the exposed workers, FEV_1_ was lower than predicted (95.6-97.8 %) and forced vital capacity (FVC) was higher (100.6-102.1%). Those differences were statistically significant, but probably not clinically significant. Cumulative smoking history (packyears) was the only parameter significantly associated with reduced FEV_1_ (*p* <.01), and no study parameter was associated with FVC. Level of MMA exposure was not associated with lung function once smoking habits were accounted for.

The high participation rate (89% of eligible workers) lends strength to the study findings, but conclusions are limited by the use of spirometry at only one point in time, and the lack of a concurrent control group.

The HSE criteria document referred to a follow-up study that included former employees, but we have not been able to document or obtain such a study. In response to inquiries, one of the authors wrote: “I can't remember if we did manage to get the second part done, we found very little as you will see from the first report” (Niven, 2009).

*Dental technicians.* Rajaniemi and Tola (Rajaniemi and Tola, 1985) reported the prevalence of symptoms and medical history in 202 Finnish dental technicians exposed to MMA. Principal concerns of the study were dermatitis and neuropathy, but respiratory effects were also considered. The dental technicians included 77 females (ages 17-54 years) employed an average of >6 years (range: 1-23 years) and 125 males (ages 17-58 years) employed an average of >8 years (range: 1-40 years). Findings were compared to 91 dental students (34 females, ages 18-51 years and 57 males, ages 15-33 years).

Self-reported information was obtained using a mailed questionnaire (91% response rate): 81% (164/202) of the dental technicians reported handling MMA “daily without skin protection,” 57% (115/202) reported handling MMA for >l-3 hours per day, 41% (83/202) handled MMA < 1 hour per day, and 4 reported no MMA exposure. MMA exposure was not described in the students.

Asthma “diagnosed by a physician” during the previous 12 months was reported by 3.5% of technicians (7/202) and 1% (1/91) of students. Among technicians, the rates of self-reported “allergic rhinitis” (31%, 63/202) and “allergic conjunctivitis” (11.4%, 23/202) were more than twice the rates reported by the students (13%, 12/91 and 5.5%, 5/91, respectively). Technicians with self-reported “dermatitis” were significantly more likely to report “symptoms of allergic rhinitis” (26% versus 42%, *p* <.O5) and “symptoms of allergic conjunctivitis” (7% versus 20%, *p* <.O5), but there was no increased prevalence of physician-diagnosed asthma.

This study is limited by its reliance on unverified self-reported information and lack of exposure data.

Nishiwaki et al. (Nishiwaki et al., 2001) assessed the health of 19 male dental technicians (mean age: 37.8 ± 12.4 years) routinely exposed to MMA. Time-weighted average MMA levels ranged from 0.16 to 4.38ppm (mean: 1.4 ppm) for an unspecified duration. Maximum MMA levels, measured over a 1.5-hour duration during hot-curing of resins, ranged up to 37.7 ppm. Post-shift urine methanol levels averaged 4.21 mgper g creatinine (range: 0.87-14.03), but duration of exposures was not reported^1^. Findings were compared to nine male dental technicians (mean age: 40.0 ±10.1 years) who worked in the same laboratory, but were not exposed to MMA (confirmed via passive sampling). Selection criteria were not otherwise specified.

Examinations included a self-administered questionnaire for symptoms, smoking habits, past and present illnesses, and daily job requirements; spirometry was performed once. Exposed and controls technicians were similar on all demographics, but nearly twice as many exposed technicians were current-smokers (57.9% versus 33.3%). Compared to controls, exposed technicians reported significantly higher prevalence of “cough during work” (6/19 versus 0/9, *p* <0.05).That difference was no longer significant after stratification by smoking. Both groups had a similar prevalence of “phlegm during work” (5/19 versus 2/9), which was reported only by smokers.

Spirometry was performed and evaluated according to recommendations of the Japan Society of Thoracic Diseases. Compared to controls, exposed technicians had significantly lower mean values of %FVC/HT 94.4% ± 7.6% versus 105.5% ± 9.1%) and %FEV_1_/HT (96.8% ± 7.2% versus 107.2% ±11.4). The only comparison that remained significant after adjustment for smoking was %FVC/HT in nonsmoking exposed versus nonsmoking controls (92.9% ± 8.8% versus 110.0% ± 3.5%, *p* <.O5).

The study is limited by its small sample size, use of spirometry at only a single time point, higher frequency of smokers in the exposed group, and failure to adjust for exposures to dusts and metals that the authors noted to be present.

*Other industries.* Marez et al. (Marez et al., 1993) evaluated the effects of MMA on the lung function of 40 French workers (mean age 37 years) exposed to MMA for >5 years (32 workers for >10 years). Findings were compared to 45 controls who had similar jobs, but had not been exposed to MMA or other known respiratory irritants. Workers with previous occupational exposure to “dusty trades” (n = 81) or short-term, low-level MMA exposures (<5 years at 0.7-2.7 ppm, *n=* 14) were excluded. The workers were employed at two factories (industries not described) where 8-hour TWA MMA concentrations were 18.5 ppm (range: 9-32ppm) and 21.6ppm (range: 11.9-38.5ppm). There were no differences in mean age, height, or weight between exposed and controls. Smokingrates were slightly higher in exposed workers (40% current-smokers, 27.5% ex-smokers) compared to controls (40% current-smokers, 15.5% ex-smokers), but cumulative smoking intensity was lower in the exposed workers (13 versus 16 pack-years).

An administered questionnaire elicited self-reported symptoms (cough, sputum production, dyspnea, wheezing) and history of asthma or chronic bronchitis. None of the workers reported a history of asthma. Symptoms were more frequent in the MMA-exposed group, but the only significant difference was chronic cough (p=.04), which remained significantly elevated after adjustment for smoking status (p=.O3). Spirometry was performed twice, at the start of a work shift ("pre-shift") and two hours before the end-of-shift ("post-shift"), according to criteria that were “stricter than” ATS recommendations (ATS, 1979, 1987). Pre-shift results did not differ between exposed and control workers. Nearly all workers had a decrease across the work shift, which was greatest among the MMA-exposed workers; the only significant difference involved mid-expiratory flow rates.

A subsequent HSE Criteria Document (Cary, 1995) questioned the validity of this study's exposure measures in light of reports that the technique used was “inaccurate."

Lindberg et al. (Lindberg et al., 1991) conducted a pilot study for the Swedish National Institute of Occupational Health to evaluate the health effects of chronic high-level exposure to MMA in 10 Swedish “floor layers” with mean age of 31 years (range: 20-60 years) who had been coating concrete floors “exclusively” with MMA-based plastics for an average duration of 4.6 years (range: < 1-12 years). The study focused on respiratory and nervous system effects. “Portable sampling equipment” was used to measure MMA levels under a variety of conditions that varied by stage of work, room size, and ventilation conditions. Daily mean concentrations of MMA varied from 62 to 601 ppm (median: 175 ppm).

Examinations included an interview regarding work-related respiratory symptoms, medical history, smoking, alcohol consumption, and routine physical examination. Findings in the exposed workers were compared to 10 age-matched controls who had participated in an earlier project. Lung function was assessed using spirometry, serial PEFR measured three times per day for 1 week, methacholine challenge, and carbon monoxide diffusing capacity (DLco).

Workers complained of acute eye and throat irritation. Three (30%) reported eye irritation at least once per week and another three (30%) reported eye irritation at least one to three times per month. Three workers complained of throat or nose irritation. In six of the workers, physical examination revealed erythema of palate arches and tonsils.

Spirometry results and DLco were within or above the normal range. All methacholine tests were normal. PEFR was performed in five workers and all were “normal"; no explanation was provided for those who did not perform PEFR. Complete blood counts were performed in 9 of 10 workers. Two had slightly elevated total white blood cell (WBC) counts (13.1 × 10^9^/L and 10.3 × 10^9^/L), a third had increased eosinophils (13%), a fourth had slightly increased monocytes (12%), and a fifth had borderline increased lymphocytes (46%).

This study is limited by its small sample size.

### Mortality studies

The mortality experience of MMA-exposed workers has been evaluated in four studies that considered nine separate cohorts. Three of those studies specifically considered mortality rates due to asthma in seven separate cohorts.

Collins et al. (Collins et al., 1989) evaluated the mortality experience of 2671 men, of whom 1561 were exposed to “significant amounts of MMA” at two plants between 1951 and 1974. The 1561 exposed workers were compared to the unexposed workers; however, the report indicated a total of 1971 unexposed workers, or 861 more than were otherwise accounted for. The authors reported a nonsignificant increase in mortality from “all respiratory disease” (standardized mortality ratio [SMR]: 2.16, observed = 4, expected=1.9, *p >* .05). Asthma-related deaths were not reported.

Walker et al. (Walker et al., 1991) evaluated mortality in three cohorts of white US male MMA manufacturing workers (Early Bristol [EB]: n = 3934, 1933-1986; Later Bristol [LB]: n = 6548, 1946-1982; Knoxville [K]: n = 3381, 1943-1982) as compared to white US males, adjusted for age and calendar period. Asthma-related deaths were less than expected overall (observed = 3, expected = 5.53) and SMRs were less than expected in all three cohorts (EB: SMR = 0.33; LB: SMR = 0.87; K: SMR = 0.73).

Lucas (Lucas, 1995) evaluated the mortality experience of 1808 white US males exposed to MMA >90 days while manufacturing acrylic sheet during 1961-1987. Cause-specific morality rates were compared to rates for white male residents of Maine. MMA-exposed workers experienced lower than expected SMR for nonmalignant respiratory disease ("bronchitis, emphysema, asthma") SMR: 63 (95% CI: 17.2-161.3) based on 4 observed versus 6.35 expected. Only limited smoking information was available.

Tomenson et al. (Tomenson et al., 2000) evaluated the cause-specific mortality rates of 4324 workers (119,822 person-years) in three cohorts of workers exposed to MMA at two UK plants while manufacturing acrylic sheets during 1949-1988. There was a lower than expected mortality rate for asthma (observed = 1, expected = 4.45; SMR not calculated).

### Case reports

#### Background

Unlike epidemiological studies, which largely depend on self-reported questionnaire data and workplace screening tests, most of the case reports and case series summarized below presented detailed personal histories and results of specific diagnostic testing. Thus, case reports can provide objective bases for specific diagnoses and they may contain the sorts of information necessary to differentiate sensitizer versus irritant effects.

In some case reports, respiratory sensitization and respiratory irritation have been differentiated on the basis of disease latency. Induction of sensitization is usually associated with a latency of weeks to months after first exposure; accordingly the onset of symptoms weeks or months after first exposure suggests sensitization. After sensitization has occurred, however, response to exposures (i.e., “elicitation") develops within minutes or hours. Irritant-induced effects generally occur without latency, i.e., onset occurs immediately after a large initial exposure (Bernstein et al., 2006b; Wallace et al., 2008). Because it can be difficult to determine whether a response immediately following exposure represents irritation-induced effects or response elicitation in a previously sensitized individual, it may be difficult to decide whether a particular reaction represents sensitization or irritation. Moreover, irritant-induced effects may have apparent latency. Initial reports of irritant-induced asthma (e.g., reactive airway dysfunction syndrome) described almost immediate onset of effects after high-level exposure (Boulet, 1988; Brooks et al., 1985), but more recent reports describe cases in which effects developed following repeated exposures over months or years (Gautrin et al., 2006; Glindmeyer et al., 2003; Kipen et al., 1994; Tarlo and Broder, 1989).

In addition, relatively low levels of irritants can provoke typical clinical signs of asthma (e.g., wheeze, chest tightness, cough, breathlessness) in individuals with nonspecific bronchial hyperreactivity (NSBH) (Gautrin et al., 2006; Kimber et al., 2007). NSBH is a characteristic of asthma caused by respiratory sensitization, but it is also found after high-level irritant exposure and as a consequence of smoking and respiratory infections (Cerveri et al., 1989; Crapo et al., 2000; Empey et al., 1976; Jensen et al., 1998). In individuals with NSBH, the clinical effects and latency pattern of sensitizers and irritants may be indistinguishable. Further confusion can result because some compounds, such as MMA, have both allergenic and irritant activities. In some cases, both mechanisms act simultaneously (Arts et al., 1998; Briatico-Vangosa et al., 1994; Kimber et al., 2007; Pauluhn and Mohr, 2005).

Immunoglobulin E (IgE) levels have been proposed as a means of differentiating respiratory sensitization and respiratory irritation. IgE production is an important and perhaps essential aspect of the process leading to respiratory sensitization (Kimber and Dearman, 2002) and measurements of total and antigen-specific serum IgE levels are standard components of the clinical evaluation of asthma and rhinitis (AHRQ, 2002; Bousquet et al., 2008; Lemiere et al., 2006; Quillen and Feller, 2006; Tarlo et al., 2008). But, unlike HMW respiratory sensitizers, which are strongly associated with specific IgE antibody production, IgE has not been consistently associated with most LMW respiratory sensitizers (Bernstein et al., 2008; Tarlo et al., 2008). In a recent meta-analysis of 11 studies that reported sensitivity and specificity for OA of specific IgE antibodies versus SIC, pooled sensitivity for LMW asthmagens was only 31.2% (95% CI: 22.9-40.8%); by contrast, pooled sensitivity for HMW sensitizers was 73.7-81.7% (Beach et al., 2005). The pooled specificity for LMW asthmagens was 88.9% (95% CI: 84.7-99.2%). Accordingly, antigen-specific IgE testing is “usually not indicated” for asthmatics exposed to LMW chemicals (Tarlo et al., 2008). Total serum IgE levels are said to have “poor predictive value” for allergic rhinitis and should not be used for diagnostic purposes (Dykewicz et al., 1998; Ng et al., 2000; Weed and Hursting, 1998). Measurement of antigen-specific IgE has been recommended in the evaluation of rhinitis patients, but most reports describe results for only HMW sensitizers (Dykewicz et al., 1998). It is not known whether the failure to detect specific IgE antibodies to LMW sensitizers reflects alternative immu-nological mechanisms or limitations of current analytical methods (Campo et al., 2007). Because test antigens for LMW sensitizers are usually prepared and evaluated in individual research laboratories, the antigens have not been standardized and results from different laboratories are usually not comparable (Bernstein et al., 2008).

Other tests used to evaluate respiratory sensitization include eosinophil counts in blood or sputum and skin prick testing (SPT). A recent meta-analysis identified only two studies that reported the sensitivity of blood eosinophil counts for LMW asthmagen-induced OA; pooled sensitivitywas 53.1% (95% CI: 10.3-91.8%) (Beach et al., 2005). The utility of eosinophil counts in allergic rhinitis has not been well studied, but is likely to be low; eosinophil counts >5-20% have been reported in up to 33% of patients with nonallergic rhinitis (Dykewicz et al., 1998; Ellis and Keith, 2006; Quillen and Feller, 2006). SPT is sometimes used as a preferred, albeit indirect measure of IgE (Quillen and Feller, 2006; Wallace et al., 2008). A recent meta-analysis identified 16 studies of asthmatics that reported results of SPT for LMW asthmagens. In five studies reporting both the sensitivity and specificity of SPT versus SIC, the pooled estimate of sensitivity was 72.9% (95% CI: 59.7-83.0%), whereas pooled estimated of specificity was 86.2% (95% CI: 77.4-91.9%) (Beach et al., 2005); sensitivitywas lower in studies not reporting specificity. SPT is a widely used clinical test in the diagnosis of allergic rhinitis, with a sensitivity that is similar to that for asthma (Wallace et al., 2008). In this context, it is a standard test for HMW sensitizers, but not LMW asthmagens (Bernstein et al., 2008).

SIC is considered the “gold standard” for confirmation of OA (Maestrelli et al., 2006; Tarlo et al., 2008; Vandenplas et al., 2006). Patients are exposed to the suspected agent(s) in an exposure chamber or workplace simulation and their respiratory response determined by spirometry or PEFR. SIC is clinically indicated for sensitizer-induced asthma, but not for irritant-induced asthma (Tarlo et al., 2008; Vandenplas et al., 2006; Vandenplas and Malo, 1997). The sensitivity and specificity of SIC have not been determined because there are no other tests to which it can be compared, but it is known to yield false-positive and false-negative results. False positives are of particular concern when the test chemical is an irritant (Beach et al., 2005; Tarlo et al., 2008; Vandenplas et al., 2006). Individuals with NSBH can have immediate bronchial responses during SIC, especially with irritant chemicals, so that responses to irritants and sensitizers cannot be distinguished (Briatico-Vangosa et al., 1994; Nicholson et al., 2005; Tarlo et al., 2008; Tarlo and Broder, 1989; Vandenplas et al., 2006; Vandenplas and Malo, 1997).

A different concern is that many of the methacry-late-containing materials that have been used for SIC challenges are mixtures; in some cases their actual compositions are not known. In such cases, positive SIC can serve to diagnose OA, but it cannot determine the causal agent.

#### Overview

The following published case reports describe 19 individuals who were evaluated because of suspected respiratory sensitization to MMA monomer and/or MMA polymer (pMMA). They have been grouped according to their sources of exposure: *dental exposures, orthopedic exposures,* and *other exposures* (Table 4). Clinical evaluations varied across reports and descriptions of testing methods were often incomplete. In most reports, exposures of concern were incompletely orwrongly described. As discussed above (see Mixtures and Cross-Reactivity), methacrylate-containing dental, orthopedic and other materials are almost always mixtures of methacrylates and acrylates plus inhibitors, activators, and other reactive compounds that are generally incompletely identified. Some authors referred to “MMA” when they meant “pMMA” or “methacrylate-containing copolymers.” Materials used for SIC were mixtures that were neither analyzed not completely described. Actual workplace exposure levels associated with sensitization and levels experienced during SIC were almost never detailed.

**Table 4:** Summary of 19 case reports.

Author	Year	Occupation/exposure context	Diagnosis or “clinical description”	PFT	PEFR	NSIC	Specific inhalation challenge
**Dental exposures**							
Lozewicz	1985	Dental Assistant: working on dental prosthetic trays	Asthma	NR	(-)	NR	(+) pMMA powder & MMA liquid NR: Placebo
Basker	1990	Denture wearer	Asthma	NR	(-)	NR	NR (see text)
Savonius	1993	Dental Technician	Asthma	NR	NR	NR	(+) Mix 10 g methacrylate powder & 10 ml methacrylate liquid NR: Placebo
Wittczak	1996	Dental Technician	Asthma	(+)	NR	NR	(+) MMA-containing acrylics (Superacryl, Duracryl)(-) Placebo
Piirila	1998	Dental nurse	Asthma	(-)	(+)	(+)H	(+) Paladur dental composite resin (-) “Scotchbond” primer NR: Placebo
Piriila	1998	Dentist	Laryngitis	(-)	(-)	(-)H	(-) Paladur or Forestacryl dental composite resins NR: Placebo
Scherpereel	2004	Dental Technician trainee	Hypersensitivity pneumonitis	(+)	NR	NR	NR
Scherpereel	2004	Dental Technician trainee	Hypersensitivity pneumonitis	(+)	NR	NR	(+) “Aerolized” MMA particles
Thorette	2006	Dental student	Hypersensitivity pneumonitis	NR	NR	NR	NR
**Orthopedic exposures**							
Lee	1984	Operating Room Anesthetist: bone cement	“Tightness in the chest”	NR	NR	NR	NR
Scolnick	1986	Surgical Nurse trainee: near bone cement mixing table	“Difficulty in breathing”	NR	NR	NR	NR
Pickering	1986	Surgical Nurse: mixing bone cement	Asthma	(-)	NR	NR	(+) Palacos bone cement with gentamicin NR: Placebo
Reynaud-Gaubert	1991	Surgical Nurse: mixing bone cement	Asthma	(-)	NR	(+)A	(+) Palacos bone cement with gentamicin
Kirby	2003	Operating Room Radiology Technician: bone cement	Bronchospasm	NR	NR	NR	NR
**Other exposures**							
Kennes	1981	Handyman: Plexiglass cutter	Asthma	(+)	NR	NR	(+) TDI varnish (+) Plexiglass shavings (-) Other (see text)
Lozewicz	1985	Railway cable joiner	“Headache, sweating, and lassitude”	NR	(+)	(-)H	(-) Acrylic cold curing resin system containing MMA (-) Placebo
Savonius	1993	Plate engraver	Asthma	NR	NR	(-)H	(+) MMA-containing glueNR: Placebo
Savonius	1993	Hearing aid assembler	Asthma	NR	NR	(+)H	(-)Ground piece of methacrylate NR: Placebo
Wittczak	2003	Secretary: photocopying	Asthma	NR	NR	(+)H	(+) MMA, heated (+) Photocopy toner (-) Placebo

Key:Note.

PFTs: = pulmonary function tests (spirometry);

PEFR: = peak expiratory flow rates;

NSIC: = non-specific inhalation challenge;

NR: = tests were either not performed or results were not reported;

H: = histamine challenge;

A: = acetylcholine challenge;

(-): = test results were normal;

(+): test results were positive (as reported by authors).

The summaries below employ the following conventions: (1) Unless noted in the summary, the composition of MMA-related materials was *not* described in the report. For those reports, the summaries either present the actual phrase used to describe the material (in “quotation marks") or simply refer to MMA-containing material. (2) Unless noted in the summary, historical exposures levels and exposure levels occurring during SIC were *not* described in the report. (3) Unless noted in the summary, the following clinical tests were *not* described in the report: (a) total IgE, (b) antigen-specific IgE; (c) eosinophil count; (d) SPT; (e) patch test for acry-late sensitizers; (f) bronchoalveolar lavage (BAL).

#### Dental exposures

Lozewicz et al. (Lozewicz et al., 1985) reported seven cases of occupational asthma, of whom two had been exposed to MMA-containing materials. One of those patients, Patient 6, was a 40-year-old male dental assistant who had worked for several years on dental prosthetic trays before he experienced work-related symptoms of chest tightness, dyspnea, and cough that persisted for several hours after mixing “pMMA powder with MMA liquid.” He gave no history of wheeze or breathing difficulty other than the work-related episodes. Spirometry and nonspecific inhalation challenge (NSIC) were not reported and PEFR away from work was normal. SIC was positive (24% fall in PEFR which resolved within 2 hours) after simulated workplace exposure mixing “PMMA powder with MMA liquid” monomer for 20 minutes. Repeat testing 1 week later resulted in a similar response. Placebo testing was not performed.

Basker et al. (Basker et al., 1990) reported a 65-year-old woman with a 12-year history of asthma, for which she had been hospitalized and chronically treated with corticosteroids. Her asthma began shortly after she was first fitted with acrylic dentures. She had had asthma attacks when exposed to cigarette smoke, petrol fumes, and perfume and she described wheezing and skin reactions after contact with acrylic fabrics. Spirometry and NSIC were not reported, PEFR was negative. She also complained of throat burning, sore mouth, and facial pain. IgE test and radioallergosorbent test (RAST) NOS were “normal.” Her asthma “subsided gradually” after she stopped wearing her dentures.

SIC were not performed, but the patient was challenged with dentures made of three different base materials: vulcanite; Flexiplast (nylon 12); and “clear heat-cured pMMA.” The first two caused no adverse effects, but 4 hours after insertion of the pMMA denture she experienced a “severe asthma attack” that required corticosteroids.

Savonius et al. (Savonius et al., 1993;) reported findings in 3152 patients evaluated for occupational respiratory disease (pneumoconiosis not included). MMA exposure was associated with 3 of 880 cases diagnosed as having OA/respiratory disease. One of those three was a dental technician.

Patient M-3 was a 46-year-old female who first developed paresthesia of the ulnar sides of both hands, but no dermatitis after 20 years working as a dental technician. Subsequently she developed “tickling in her throat, yawning, cough, tiredness and chest tightness.” Symptoms subsided during vacation and reappeared within one week after return to work. SPT ("methacrylate and PEGDMA") and patch tests (acrylate series) were negative. Spirometry, PEFR, and NSIC were not reported. SIC was positive (PEFR fell 26%; immediate and late reaction) with 30 minutes of work simulation mixing “methacrylate powder” and “methacrylate liquid."

Wittczak et al. (Wittczak et al., 1996) described a 40-year-old female dental technician who experienced increasingly severe attacks of coughing and nasal secretions beginning several hours after contact with MMA-containing materials ("Superacryl, Duracryl"). The attacks first began 6-8 months after she started working and responded to cromolyn and corticosteroids, which allowed her to continue working. After nearly 13 years, she received a disability pension because of OA. Six years later, she was hospitalized for respiratory distress after again working with MMA-containing prosthetic materials. Total IgE was 107.6 kU/L, the peripheral blood eosinophil count was elevated (935 per mm^3^), SPT was negative (common allergens) and acrylate patch tests were negative. Spirometry was abnormal (FVC: 75% predicted, FEV1: 82% predicted). PEFR and NSIC were not reported.

SIC was positive *(FEV_1_* fell >40% at 4 hours and PEFR fell > 50% at 24 hours) after work simulation mixing “liquid MMA” and “MMA powder” for 20 minutes. Twenty-four hours following SIC, nasal lavage showed increased WBC count (311.6× 10^3^/ml) and increased eosinophil count (54.7 × 10^3^/ml).

Piirila et al. (Piirila et al., 1998) described 12 dental workers with evidence of “acrylate induced respiratory hypersensitivity.” Of the 12, only 2 had been exposed to MMA-containing materials.

Patient 6 was a 51-year-old female dentist with a clinical diagnosis of occupational laryngitis. She had been exposed to acrylics for 27 years and had “symptoms” NOS for 23 years. Details of her work exposures to methacrylate-containing materials were not reported. Total IgE was 17 kU/L. SPT was negative for common environmental allergens, latex, and methacrylates (MMA, 2-HEMA, bis-GMA, EGDMA, TREGDMA) and acrylate patch tests were negative. Spirometry PEFR and NSIC were normal. SIC was negative after workplace simulation with “prosthesis dose of powder” (Paladur®, Forestacryl®).

Patient 8 was a 48-year-old female dental nurse with a 27-year history of “mixing dental composite resin products” that contained “various acrylates.” During the prior 2 years she experienced hoarseness, sore throat, nasal stuffiness and dyspnea, particularly when preparing orthodontal fixatives. Total IgE was increased (203 kU/L). SPT was negative for common environmental allergens, latex, and methacrylates (MMA, 2-HEMA, bis-GMA, EGDMA, TREGDMA) and acrylate patch tests were negative. Spirometry was normal, histamine inhalation challenge testing was “slight positive” (FEV1 fell 15% with 0.48 mg histamine), and PEFR was positive. SIC was positive after workplace simulation exposure to Paladur® ("mucosal changes and symptoms of the upper respiratory tract"; rhinomanometry: 70% increase in airflow resistance; FEV_1_ fell only 6%; PEFR fell 20% after 16 hours). SIC was negative after workplace simulation exposure to Scotchbond® primer (2-HEMA and bis-GMA).

Scherpereel et al. (Scherpereel et al., 2004) reported two cases of “hypersensitivity pneumonitis” in dental technician trainees “within the first weeks of exposure to MMA” in a laboratory.

Patient 1 was a 24-year-old female who was hospitalized for progressive severe dyspnea and cough 6 months after the start of training. Physical examination revealed diffuse bilateral crackles, arterial blood was hypoxemic (P_a_o_2_: 55 mm Hg), DLco was 45% of predicted, and computed tomography (CT) scan showed ground glass pattern. Her symptoms responded to systemic corticosteroids treatment. One month later, after returning to work for 3 days, she again required hospitalization for severe dyspnea. She was hypoxemic (P_a_o_2_: 58 mm Hg). Spirometry was abnormal (FVC: 50% predicted; FEV1 24% predicted). BAL was abnormal (cell count: 570,000 cells/ml; 10% macrophages, 60% lymphocytes, 25% neu-trophils). PEFR, NSIC, and SIC were not performed.

Patient 2 was a 20-year-old female who was hospitalized for acute respiratory distress a few weeks after the start of training. She had “major dyspnea,” cough and dif-fusebilateral crackles, arterialbloodwas hypoxemic (P_a_o_2_: 65 mm Hg), she had “pulmonary diffusion abnormality,” and chest X-ray showed bilateral ground glass patterns. Spirometry was abnormal (total lung capacity [TLC]: 67% of predicted). PEFR and NSIC were not performed. Her symptoms responded to systemic corticosteroids treatment. SIC was positive ("moderate dyspnea,” 20% fall in TLco, 20% fall in DLco, and 30% lymphocytes in bron-choalveolar lavage [BAL]) after exposure to “aerolized particles of MMA” while in a “glass cage."

Thorette et al. (Thorette et al., 2006) reported a 19-year-old female prosthetic dentistry student who developed hypersensitivity pneumonitis after working with “MMA.” No other clinical details were described. CT scan revealed micronodules and ground glass infiltrates. BAL was positive BAL BAL was positive ("hypercellular-ity,” >50% lymphocytes) and “...alveolar macrophages filled with methacrylate particles.” Spirometry, PEFR, NSIC, and SIC were not reported.

#### Orthopedic exposures

Lee (Lee, 1984) reported a 25-year-old female anesthetist who experienced headache and chest tightness during her first operating room exposure to “bone cement vapor.” She was exposed to the “same substance” 6 months later and developed headache, dizziness, palpitation, tightness in the chest, and erythema on her face, arms, and neck. Her vital signs were immediately abnormal (blood pressure [BP]: 210/110 mm Hg; heart rate [HR]: 185 beats/ min; respiratory rate [RR]: 36/min), but normalized within 30 minutes and she recovered “without complications.” No testing was performed.

Scolnick and Collins (Scolnick and Collins, 1986) reported a 31-year-old female trainee scrub nurse who experienced “bifrontal headache, nausea and lighthead-edness “during her first two orthopedic procedures” utilizing “Surgical Simplex P” bone cement (reported to contain 97% MMA monomer and 3% additives such as N,N-dimethyl-p-toluidine and hydroquinone). “Within minutes” of her third exposure to Simplex P, she developed diffuse erythroderma, dyspnea, hypertension, headache, and diffuse neurological symptoms. She remained in the operating room for about 30 minutes, standing within 2 feet from the orthopedic cement mixing bowel, before seeking medical attention. Her initial vital signs were abnormal (BP: 180/100mm Hg; HR: 120 beats/min; RR: 26/min), which normalized within 30 minutes after receiving subcutaneous epinephrine. Spirometry, PEFR, NSIC, and SIC were not performed. On 2 subsequent days, air sampling for MMA in the operating room “near the mixing table during an operation” reported MMA levels of 0.4-1.5ppm monitored over 15-minute periods (sampling method not described).

Pickering et al. (Pickering et al., 1986) reported respiratory effects in a 56-year-old female orthopedic surgical nurse who developed cough, wheezing and breathlessness shortly after beginning use of Palacos® R bone cement “in which copolymer methyl acrylate and gentamicin are mixed with MMA.” Over a period of more than 11 years, she had repeatedly worked with other MMA-containing bone cements (e.g., CMW®) without experiencing similar respiratory effects. Her symptoms resolved when she went on holiday and “recurred immediately” when she returned to work. Physical examination was not described. Total IgE was 218 kU/L and “skin tests” to “common allergens” were negative. Spirometry was normal; PEFR and NSIC were not reported.

SIC was positive (FEV1 fell 25% at 6 hours) after workplace simulation “identical with the normal procedure in the operating theatre,” but not during a placebo test. During SIC, MMA levels were measured by infrared gas analyzer; MMA levels reached 374 ppm during the first 2 minutes when bone cement was mixed on an open trolley and 76 ppm when it was mixed in a fume cabinet.

Reynaud-Gaubert et al. (Reynaud-Gaubert et al., 1991) reported OA in a 39-year-old female orthopedic nurse with a history of seasonal rhinitis and conjunctivitis who developed asthma after working for an unstated duration with Palacos® bone cement containing gentamicin. SPT was positive for common allergens (e.g., mites, feathers, and pollen). Spirometry was “normal,” inhalation challenge with acetylcholine was “positive...a typical asthmatic response” and PEFR was not performed. SIC was positive (25% fall in FEV_1_ after 30 minutes) after work simulation with Palacos® and “liquid MMA.” Asthma did not recur after the Palacos® with gentamicin was replaced by a different brand of bone cement (not identified).

Kirby et al. (Kirby et al., 2003) reported a 48-year-old female radiology technologist with a history of asthma, but no prior exposure to MMA or pMMA, who experienced chest tightness while in an operating room where bone cement was being mixed. (The report described exposure to “pMMA vapors.") Symptoms improved within 5 minutes of leaving the room and worsened upon her return. She was referred to an emergency room where she had shortness of breath, wheeze, watery eyes, and rhinorrhea. Her vital signs were BP: 132/90mm Hg; HR: 100 beats/min; RR: 24/min. Spirometry, PEFR, NSIC, and SIC were not reported.

#### Other exposures

Kennes et al. (Kennes et al., 1981) reported a 33-year-old male smoker who developed dry cough, rhinitis, and asthma after 6 months of working and living on site in a factory that manufactured electrical equipment. His jobs included cutting Plexiglas plates, manipulating phospho-rescentproducts, solderingaluminum, and spraying epoxy resin paints. On physical examination he had obstructive rhinitis with polyposis, wheezing, and severe breathless-ness. Spirometry showed “marked obstructive airways disease, reversible after inhalation of sympathomimetic amines.” Laboratory results included blood eosinophil count of 747 cells/mm^3^; “numerous” eosinophils in the sputum; low total IgE (4 kU/L); and negative specific IgE (RAST) for common allergens, *Aspergillus fumigatus,* and *Candida albicans;* and negative “skin tests” (not otherwise specified). PEFR and NSIC were not performed.

SIC was performed for five different workplace simulations: positive (FEV1 fell 43%, FVC fell 18% at 30 minutes) after he “handled plexiglass shavings dust,” with “greater immediate and a late asthmatic response” on retesting 2 days later; positive ("immediate asthmatic response") after “painting with TDI varnish"; negative after soldering with aluminum wires, painting with epoxy resin, and handling phosphorescent powder.

Savonius et al. (Savonius et al., 1993;) reported findings in 3152 patients evaluated for occupational respiratory disease (pneumoconiosis not included). MMA exposure was associated with 3 of 880 cases diagnosed as having OA/respiratory disease. Two of those three are described below.

Patient M-1 was a 48-year-old female plate engraver who developed “respiratory distress” along with sneezing, rhinorrhea, and nasal stuffiness after 92 months of “occasionally” using MMA-containing glue. Total blood IgE was 88 kU/L; SPT (common allergens) and acrylate patch tests were negative. Spirometry, PEFR, and NSIC were not reported. SIC was positive (PEFR fell 24% 8 hours post exposure) after 30-minute work simulation spreading glue. Her symptoms persisted after the glue was replaced with cyanoacrylate glue. Symptoms persisted after she quit her job.

Patient M-2 was a 32-year-old male smoker who worked as a hearing device assembler and developed “respiratory symptoms” diagnosed as asthma after a month of daily exposure to “MMA.” Total blood IgE was 9 kU/L; SPT (common allergens) and acrylate patch tests were negative. Spirometry and PEFR were not reported; NSIC was positive ("change in the reactivity of the airways in the histamine test"). SIC was “negative” (PEFR fell 15%) after grinding “a piece of methacrylate” in an exposure chamber.

Lozewicz et al. (Lozewicz et al., 1985) reported seven cases of occupational asthma, of whom two had been exposed to MMA-containing materials. One of those patients, Patient 7, was a 52-year-old male railway cable joiner who described headache, sweating, and lassitude when working with an “acrylic cold curing resin system containing MMA,” He had smoked “for many years” and reported episodes of cough and wheeze that were “not clearly work related.” The duration of exposure before onset of symptoms was not specified. Spirometry was not reported. PEFR “indicated asthma,” there was “no fall in FEV1” following histamine inhalation challenge, and SIC was negative “using the resin.” SPT was negative for common inhalant allergens.

Wittczak et al. (Wittczak et al., 2003) reported a 44-year-old, female secretary who had a 2-year history of rhinorrhea, dyspnea, and coughing attacks that occurred 15-20 minutes after making photocopies using xerographic toner containing “polystyrene-n-butyl methacrylate, polystyrene-n-butyl acrylate, etc.” The patient reported only work-related symptoms. Physical examination and routine laboratory tests were normal. Total IgE was “low” (18.04 ku/L) and SPT (common allergens) was negative. NSIC (histamine inhalation challenge) showed bronchial hyperreactivity. Spirometry and PEFR were not reported. SIC was positive after 18 minutes of photocopying (FEV_1_ fell 24% at 1 hour) and after exposure (duration not described) to “thermally activated (80°C) MMA” (FEV_1_ fell 30% at 1 hour), but negative after exposure to thermally activated polystyrene. Nasal lavage eosinophils increased 24 hours after photocopying and “thermally activated (80°C) MMA."

## Discussion

Methyl methacrylate is a respiratory irritant and dermal sensitizer that has been associated with occupational asthma in a small number of case reports. Those reports have raised concerns that MMA might also act as a human respiratory sensitizer, a category of chemicals of much public health concern because of their potential to cause substantial morbidity. This concern is of particular importance because of the high volume of MMA production and the large numbers of workers and others who might be exposed. To better understand that possibility, this review was undertaken to update the literature and evaluate the weight of evidence that MMA causes respiratory sensitization.

Only a limited number of metabolic, in silico and in chemico, studies exist that provide insights into the respiratory sensitization potential of MMA. From a physical chemistry perspective, its relatively low electrophilicity and relatively high water solubility indicate that MMA should be expected to have low sensitizing potential as compared to other acrylates and methacrylates. Experimental data also indicate that α-methyl substitution of an ester, such as MMA, decreases its reactivity compared to corresponding unsubstituted esters, thus further supporting expectations that MMA would have comparatively low sensitizing potential. In addition, SAR studies found that esters have generally little or no activity as respiratory sensitizers and that LMW compounds with only one reactive functional group, such as MMA, pose a low respiratory sensitization hazard. Finally, SAR analyses using a model with a reported specificity of 99% for respiratory sensitizers judged MMA to be “inactive.” Taken together, these studies lend substantial weight of evidence that MMA should not be expected to cause respiratory sensitization.

The sensitization potency of MMA has only rarely been tested in vitro. In cellular response assays, MMA demonstrated weak, transient, and mainly nonspecific stimulation of cytokine production and gave no evidence that lymphocyte transformation was induced. Although limited, these study findings provide no evidence that MMA should be expected to cause respiratory sensitization.

Extensive in vivo animal testing has evaluated the capacity of MMA to cause contact skin sensitization, but such studies provide little insight to its potential for respiratory sensitization. Direct testing for inhalation sensitization in guinea pigs and mice has not been reported, but LLNA has been performed. The LLNA results indicate that MMA has low sensitizing potency, substantially less than that of agents linked to respiratory sensitization. Because the “inherent sensitizing potential” of a chemical is viewed as its most important factor for induction and elicitation of respiratory sensitization (Kimber et al., 2007), its low potency provides further reason to predict that MMA does not act as a respiratory sensitizer.

Human studies comprise a larger and broader body of evidence. We identified one cohort and five cross-sectional studies of primary MMA workers, two cross-sectional studies of MMA-exposed dental workers, two cross-sectional studies of workers exposed in other industries, and four mortality studies of MMA workers. Taken together, those studies provide evidence of respiratory irritation, but not airway sensitization or occupational asthma.

Nineteen case reports described occupational asthma, laryngitis, or hypersensitivity pneumonitis in MMA-exposed workers, but it remains uncertain whether any reflected MMA-induced sensitization. Most cases had been exposed to mixtures of LMW sensitizers and irritants, or their exposures were not fully described. Specific challenge testing was performed in 13 of the 19 cases. Of those, three had negative tests. Two others had positive SIC, but had not been tested with MMA monomer. Another three were tested at high MMA levels that would have caused significant irritation: two were tested with Palacos® in a manner previously shown to achieve levels up to 1500ppm; the third was tested with “MMA” heated to 80°C, a process that would have resulted in very high air concentrations. The final five case underwent SIC testing with “impure” mixtures of methacrylates that were not analyzed, but probably contained “many additional acrylates... additives and additional impurities” (Kanerva, 1993). Together and separately, these reports provide little evidence that MMA is a respiratory sensitizer. Due to high exposure levels that occurred during testing, it is not possible to distinguish irritation versus sensitization. And because most SIC were conducted using uncharacterized mixtures of sensitizers and irritants, it is not possible to attribute the observed effects to MMA.

The difficulty of interpreting the results of SIC conducted using incompletely characterized test chemicals and unexpectedly high exposure levels is reflected in a case report (not included above) that might have wrongly led to an inference of MMA-induced asthma. Vallieres et al. (Vallieres et al., 1977) described a spray painter with progressive rhinitis and asthma beginning one month after starting to work with a paint containing 93% MMA, 1.4% dimethyl ethanolamine (DMEA), 0.6% 1,4-dioxane, and small amounts of pigment. SIC testing was performed by spraying a liter of test liquid in a “small, poorly ventilated room” over 13-14 minutes; air levels were not measured. SIC was positive with paint (FEV1 fell 29% immediately, and 23% at 8 hours) and with a 2% aqueous solution of DMEA *(FEV_1_* fell 58% immediately, and 27% at 8 hours). By contrast, SIC was negative with 100% MMA and with a 0.6% aqueous solution of 1,4-dioxane. Skin prick tests with “high concentrations” of DMEA caused similar wheal-and-flare response in the case subject and three unexposed controls, suggesting an “irritative effect."

The Vallieres report described what might have seemed to be a “classic” asthmatic response to MMA, but was actually not MMA related. Moreover, although the SIC response to DMEA suggested a sensitizer-induced response, there are no other reports of DMEA-induced allergy; its strong irritating capacity, however, is well recognized (Klonne et al., 1987; Leung and Blaszcak, 1998; Smyth et al., 1951). And despite a relatively low vapor pressure (4 mm Hg at 20°C; New Jersey Department of Health, 2010), high air concentrations of DMEA would be expected after spraying a liter in a small, closed space. Thus, this case illustrates that without full analysis and testing of an MMA-containing mixture, it may not be possible to correctly identify the agent causing a positive SIC. And without adequate air monitoring, it may also not be possible to distinguish effects due to irritation versus sensitization.

It is notable that despite the very large numbers of MMA-exposed workers worldwide, there have been few documented cases of MMA-related occupational asthma. Moreover, none of the case reports reviewed above described workers employed in the primary MMA industries, where sophisticated industrial hygiene controls would be expected. To the contrary, all of the cases were exposed in secondary industries likely to lack adequate exposure controls: eight case reports involved dental work, five case reports involved operating room personnel, and four involved clerical and craft work. Lack of exposure data for most activities in the secondary MMA industries makes generalizations uncertain, but it seems likely that these workers were more subject to high exposures because of inadequate industrial hygiene and uncontrolled work practices. In addition, fine work such as dental procedures and nail sculpting may require that MMA-containing materials be used close to the worker's breathing zone. Thus, it would not be surprising if these workers were at risk for frequent high-level irritant exposures.

Accordingly, the weight of evidence, both experimental and observational, argues that MMA is not a respiratory sensitizer. However, there is more than sufficient evidence that it can act as a human respiratory irritant and as such, it may also cause irritant-induced occupational asthma. The distinction between the two mechanisms (i.e., irritant versus sensitizer) has important implications for workplace engineering controls and public health.

Without regard to underlying mechanisms of injury, our review also suggests important actions that should be taken to prevent adverse effects of “MMA exposure,” especially among workers in secondary MMA industries. Suppliers and producers of MMA-containing dental, surgical, and cosmetic products should provide more informative MSDS and labels, including complete descriptions of mixture components. Operating rooms and dental laboratories should be equipped with vacuum exhaust mixing bowls or negative-pressure hoods for preparation of bone cement and dental composites. Other workers using MMA-based composites should be instructed in appropriate ways to increase ventilation and minimize exposures. Finally, there is need to ensure more frequent exposure monitoring of MMA workers, including a focus on peak excursions rather than time-weighted-average exposures, which may not adequately identify the actual risks of workingwith volatile irritants such as MMA.

## Footnotes

1 The current American Conference of Industrial Hygienists (ACGIH) Biological Exposure Indice (BEI) for methanol is 15 mg/L in end-of-shift urine (ACGIH, 2010).

## Abbreviations:

ACD: allergic contact dermatitis
BAL: bronchoalveolar lavage
GHS: Globally Harmonized System of Classification and Labeling of Chemicals
HMW: high molecular weight
LLNA: local lymph node assay
LMW: low molecular weight
LNC: lymph node cells
NOS: not otherwise specified
NSBH: nonspecific bronchial hyperreactivity
NSIC: nonspecific inhalation challenge
OA: occupational asthma
PEFR: peak expiratory flow rate
SIC: specific inhalation challenge
SPT: skin prick testing
